# B cell–based therapy produces antibodies that inhibit glioblastoma growth

**DOI:** 10.1172/JCI177384

**Published:** 2024-08-29

**Authors:** Si Wang, Brandyn A. Castro, Joshua L. Katz, Victor Arrieta, Hinda Najem, Gustavo I. Vazquez-Cervantes, Hanxiao Wan, Ian E. Olson, David Hou, Mark Dapash, Leah K. Billingham, Tzu-yi Chia, Chao Wei, Aida Rashidi, Leonidas C. Platanias, Kathleen McCortney, Craig M. Horbinski, Roger Stupp, Peng Zhang, Atique U. Ahmed, Adam M. Sonabend, Amy B. Heimberger, Maciej S. Lesniak, Cécile Riviere-Cazaux, Terry Burns, Jason Miska, Mariafausta Fischietti, Catalina Lee-Chang

**Affiliations:** 1 Department of Neurological Surgery, Northwestern University, Feinberg School of Medicine, Chicago, Illinois, USA.; 2 Lou and Jean Malnati Brain Tumor Institute, Chicago, Illinois, USA.; 3 Department of Neurological Surgery, University of Chicago Medicine, Chicago, Illinois, USA.; 4 Robert H. Lurie Comprehensive Cancer Center of Northwestern University, Chicago, Illinois, USA.; 5 Department of Medicine, Division of Hematology and Oncology, Northwestern University Feinberg School of Medicine, Chicago, Illinois, USA.; 6 Department of Medicine, Jesse Brown Veterans Affairs Medical Center, Chicago, Illinois, USA.; 7 Department of Neurology, Northwestern University Feinberg School of Medicine, Chicago, Illinois, USA.; 8 Department of Neurological Surgery, Mayo Clinic, Rochester, Minnesotta, USA.

**Keywords:** Immunology, Oncology, Brain cancer, Cancer immunotherapy, Extracellular matrix

## Abstract

Glioblastoma (GBM) is a highly aggressive and malignant brain tumor with limited therapeutic options and a poor prognosis. Despite current treatments, the invasive nature of GBM often leads to recurrence. A promising alternative strategy is to harness the potential of the immune system against tumor cells. Our previous data showed that the B_Vax_ (B cell–based vaccine) can induce therapeutic responses in preclinical models of GBM. In this study, we aimed to characterize the antigenic reactivity of B_Vax_-derived Abs and evaluate their therapeutic potential. We performed immunoproteomics and functional assays in murine models and samples from patients with GBM. Our investigations revealed that B_Vax_ distributed throughout the GBM tumor microenvironment and then differentiated into Ab-producing plasmablasts. Proteomics analyses indicated that the Abs produced by B_Vax_ had unique reactivity, predominantly targeting factors associated with cell motility and the extracellular matrix. Crucially, these Abs inhibited critical processes such as GBM cell migration and invasion. These findings provide valuable insights into the therapeutic potential of B_Vax_-derived Abs for patients with GBM, pointing toward a novel direction for GBM immunotherapy.

## Abstract 2

B-cell-based therapies produce antibodies that interfere with glioma migration, invasion, and overall growth. The potential role of antibody immunity in GBM is unknown.

## Introduction

Glioblastoma (GBM) is an aggressive and malignant brain tumor that arises from glial cells (1). GBM is one of the most common and deadly forms of brain cancer in adults, with a median survival of approximately 15 months after diagnosis. The current standard of care for GBM includes surgery, radiation therapy, and chemotherapy, but the overall prognosis remains poor (2). A major obstacle in treating GBM is its remarkable ability to invade and migrate into surrounding healthy brain tissues, making complete gross total surgical resection impossible and thus leading to inevitable tumor recurrence (3). As such, there is a pressing need to explore alternative therapeutic avenues to inhibit GBM progression and improve patient outcomes.

Harnessing the immune system to modulate tumor progression and remote sites of invasion is a compelling strategy for GBM treatment (4). While most immunotherapy efforts have historically focused on T cells, the role of B cells, especially in the context of GBM, remains less explored. Recent studies suggest that B cells and their secreted Abs can influence tumor growth, metastasis, and response to treatment (5–9). The presence of tertiary lymphoid structures (TLSs) in solid cancers, containing B cells undergoing somatic hypermutation, confers a favorable prognosis (10–12). As such, our laboratory is developing an immunotherapeutic approach for cancer using activated B cells as a cell-based vaccine (B_Vax_) against GBM (13, 14). B_Vax_ is a B cell–based vaccine comprising 4-1BBL^+^ B cells activated through CD40 agonism and IFN-γ stimulation. Advantages of B cell therapy relative to other types of immunotherapies include its antigen-presenting capability (15–17), shared cognate antigen specificity with T cells (18), ability to generate tumor-reactive Abs (12), and circulatory mobility enabling tumor and secondary lymphoid organ infiltration (19, 20). Moreover, the relative ease as well as timely ex vivo activation and expansion from patient-derived circulating B cells reduces the cost of generating a personalized cell-based therapeutic. As such, B cell–based vaccines represent a promising, yet underinvestigated, immunotherapeutic approach (21–24) warranting further study in GBM.

In this study, we aimed to determine the humoral response induced by B_Vax_, assess the tumor-reactive nature of B_Vax_-derived Abs, and evaluate their therapeutic potential in preclinical models. This analysis reveals the role of B_Vax_ in the immune-tumor interplay and its therapeutic potential for patients with GBM through a blend of molecular and proteomics analyses.

## Results

### B_Vax_ differentiates into plasmablasts and harbors potentially tumor-reactive B cell receptors.

**Figure 1:**
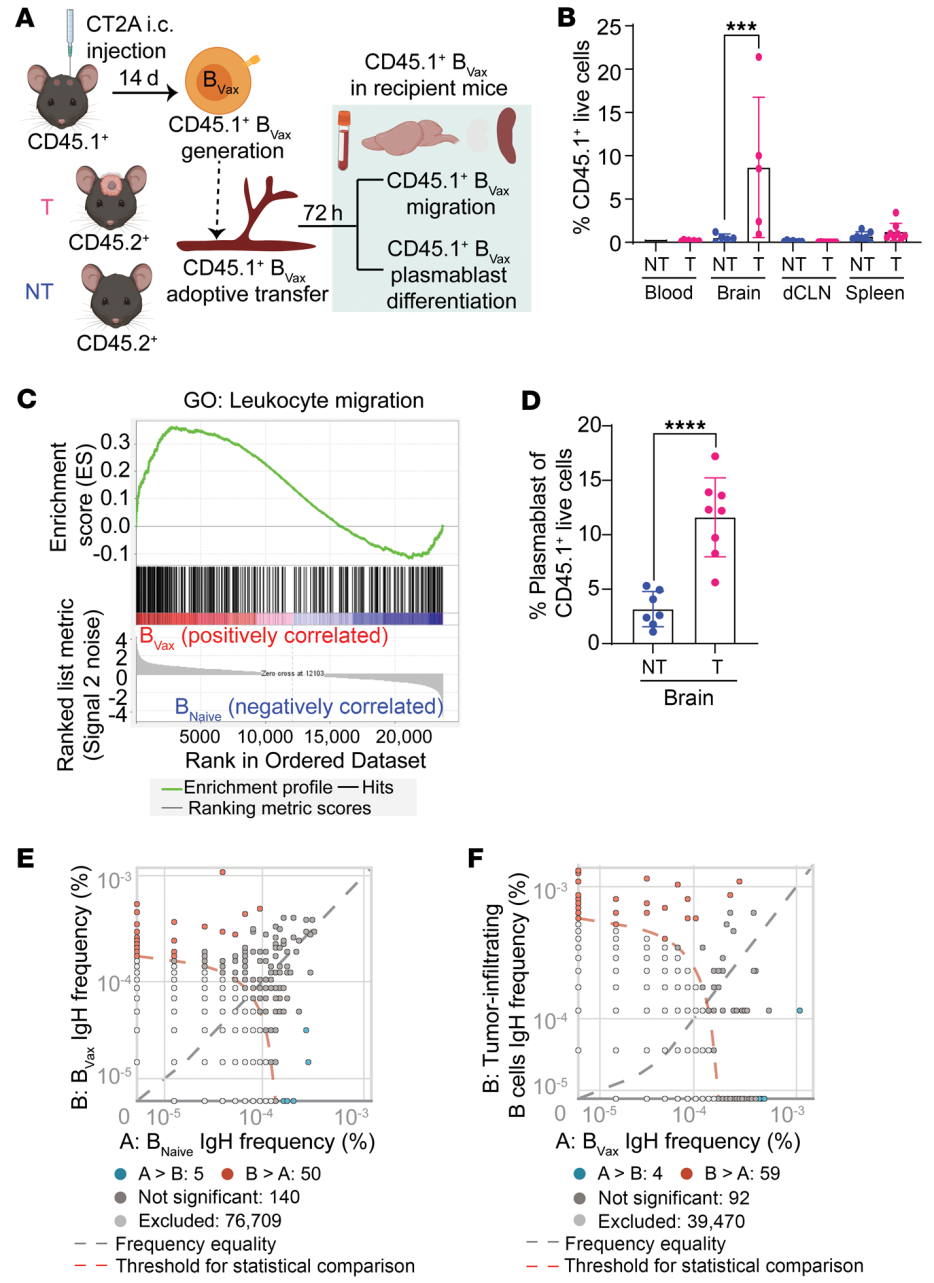
B_Vax_ differentiates into plasmablasts and generates potentially tumor-reactive B cell Igs. (**A**) Schema illustrating the experimental design to investigate the potential of B_Vax_ to migrate to the glioma and differentiate into Ab-producing cells (plasmablasts). i.c., intracranial; T, tumor; NT, nontumor. (**B**) Percentage of B_Vax_ (CD45.1^+^) cells in various tissues (blood, brain, deep cervical lymph node [dCLN], and spleen) of intracranial tumor–bearing and nontumor-bearing mice via flow cytometry (*n* = 5 for each group). (**C**) GSEA of the indicated datasets comparing the transcriptional profile between B_Vax_ and B_Naive_. Data were pooled from 3 independent experiments. (**D**) Percentage of plasmablasts (CD19^+^CD20^–^CD38^+^) within the B_Vax_ population of the brain via flow cytometry (*n* = 8 for each group). (**E**) Representative dot plot of BCR clones from BCR IgH sequencing comparing murine B_Vax_ and B_Naive_. Unique clones in B_Vax_ are shown in red; unique clones in B_Naive_ are shown in blue (*n* = 3 for each group). (**F**) Representative dot plot of BCR clones from BCR IgH sequencing comparing murine TIB cells (*n* = 2) and B_Vax_ (*n* = 3). Unique clones in TIB cells are shown in red; unique clones in B_Vax_ are shown in blue. Data are the mean ± SD. ***P < 0.001 and ****P < 0.0001, by 1-way ANOVA. NT, nontumor; T, tumor.

To determine the humoral responses generated by B_Vax_, we first analyzed the potential of B_Vax_ to migrate to the tumor and differentiate into Ab-producing cells (plasmablasts). Using the CD45.1 versus CD45.2 congenic mouse model (Figure 1A), we found that, upon intravenous injection, CD45.1^+^ B_Vax_ preferentially migrated to the glioma-bearing brains 72 hours after injection (Figure 1B). Gene set enrichment analysis (GSEA) of B_Vax_ showed upregulation of the Gene Ontology (GO) gene set involved in leukocyte migration (Figure 1C and Supplemental Table 1; supplemental material available online with this article; https://doi.org/10.1172/JCI177384DS1). The potential of B_Vax_ to differentiate into Ab-producing cells after migrating into the glioma was confirmed in vivo using CT2A tumor–bearing mice treated with B_Vax_. Approximately 10% of the B_Vax_ showed a CD38^+^CD20^–^CD19^+^ plasmablast phenotype (Figure 1D). Analysis of the B_Vax_ and B_Naive_ heavy-chain receptor repertoire (bulk IgH sequence) revealed comparable B cell receptor (BCR) repertoire diversity between B_Vax_ and B_Naive_ cells (Supplemental Figure 1A). However, B_Vax_ might present differential reactivity compared with B_Naive_ cells (Figure 1E, Supplemental Table 2, and Supplemental Table 3). Among the B_Vax_ BCRs, 92 were shared with glioma-infiltrating B cells (tumor-infiltrating B [TIB] cells, Figure 1F and Supplemental Table 4). Additionally, approximately 2% of B_Vax_ BCRs overlapped with glioma-infiltrating B cells but were absent in B_Naive_ BCRs (Supplemental Figure 1B). This suggests that B_Vax_ may harbor tumor-reactive BCRs.

### Characterization of murine B_Vax_-derived immunoglobulin reactivity.

**Figure 2:**
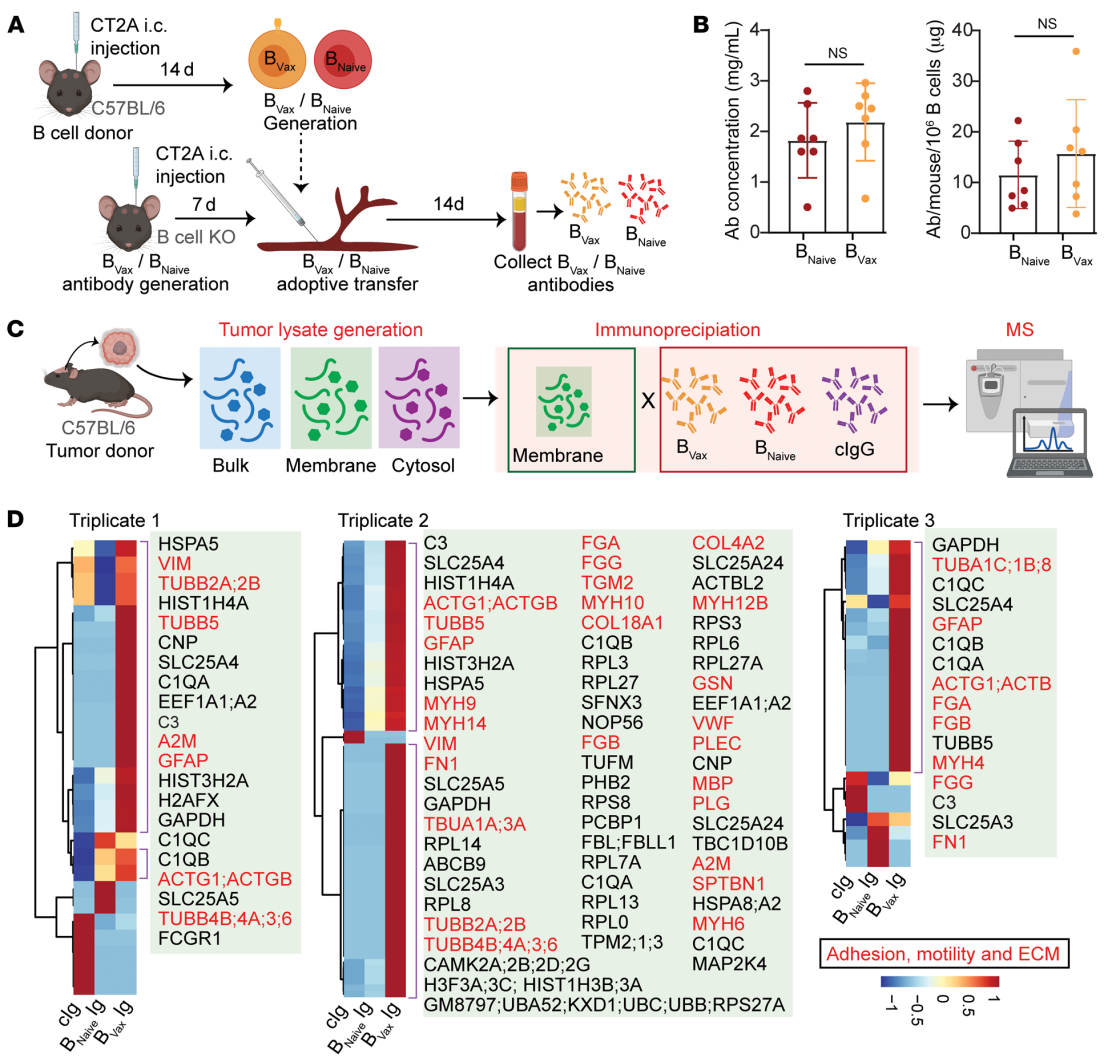
Characterization of murine B_Vax_-derived Ig reactivity. (**A**) Schema demonstrating how in vivo B_Vax_-derived Igs are produced from mice bearing CT2A gliomas. (**B**) Amount of B_Vax_-derived Igs generated from mice bearing GBM tumors ( *n* = 7 for each group). (**C**) Schema depicting the protocol for the murine IP-MS experiments used to identify tumor-specific antigens recognized by B_Vax_-derived Igs. (**D**) Heatmap revealing hierarchical clustering of GBM tumor antigens recognized by B_Vax_-derived Igs. Each triplicate corresponds to an independent IP-MS experiment. In triplicate 1, B_Vax_-derived Igs were pooled from 10 mice, and B_Naive_-derived Igs were pooled from 11 mice. In triplicate 2, B_Vax_-derived Igs were pooled from 11 mice, and B_Naive_-derived Igs were pooled from 10 mice. Triplicate 3 involved B_Vax_-derived Igs pooled from 10 mice and B_Naive_-derived Igs pooled from 12 mice. Data in **B** are the mean ± SD and were analyzed by 2-tailed Student’s *t* test.

To examine B_Vax_ antigenic reactivity, B_Vax_- and B_Naive_-derived immunoglobulins (Igs) were produced in vivo by adoptive transfer of B_Vax_ or B_Naive_ into CT2A tumor–bearing, B cell–deficient mice (Figure 2A). After 2 weeks, blood from the experimental groups (B_Vax_ and B_Naive_) was collected, and Igs were isolated using a Protein A/G Spin Column. Comparable amounts of Igs were obtained for both groups (Figure 2B). To analyze the reactivity of these Igs, we performed a immunoproteomics study using immunoprecipitation–mass spectrometry (IP-MS). Intracranial glioma homogenates were produced and fractionated into the membrane and cytosolic fractions (Figure 2C). Proteins enriched in the membrane fraction were then immunoprecipitated with the B_Vax_- or B_Naive_-derived Igs. Commercially available mouse IgG (cIgG) was used as a control. The MS analysis revealed that B_Vax_ Igs had unique reactivity compared with the B_Naive_-derived Igs or control Igs (Figure 2D and Supplemental Table 5). More specifically, the B_Vax_ produced Igs with preferential reactivity to factors involved in cell motility, extracellular matrix (ECM), and membrane organization, such as fibrinogen, fibronectin, and myosin, as shown by the GO pathway analysis (Supplemental Figure 2). To determine whether the B_Vax_ Abs specifically target ECM and membrane proteins when isolated from a more controlled environment, we conducted additional immunoprecipitation experiments using conditioned medium and cell membrane fractions from CT2A tumor cells cultured in vitro. Consistent with our initial findings, B_Vax_ Abs specifically targeted membrane (such as collagen receptor, integrin-linked kinase [ILK] complex, tight junction proteins, Rho GTPases, vinculin, and nischarin) and ECM proteins (such as gelsolin, collagens, fibrinogen, EGF-containing fibulin-like ECM protein 2, SPARC-like protein 1, matrix metalloproteinases, caldesmon, and asporin) involved in cell adhesion and motility (25–39) (Supplemental Figure 3 and Supplemental Table 6), confirming their preferential reactivity.

### Characterization of patient B_Vax_–derived Ig reactivity.

**Figure 3:**
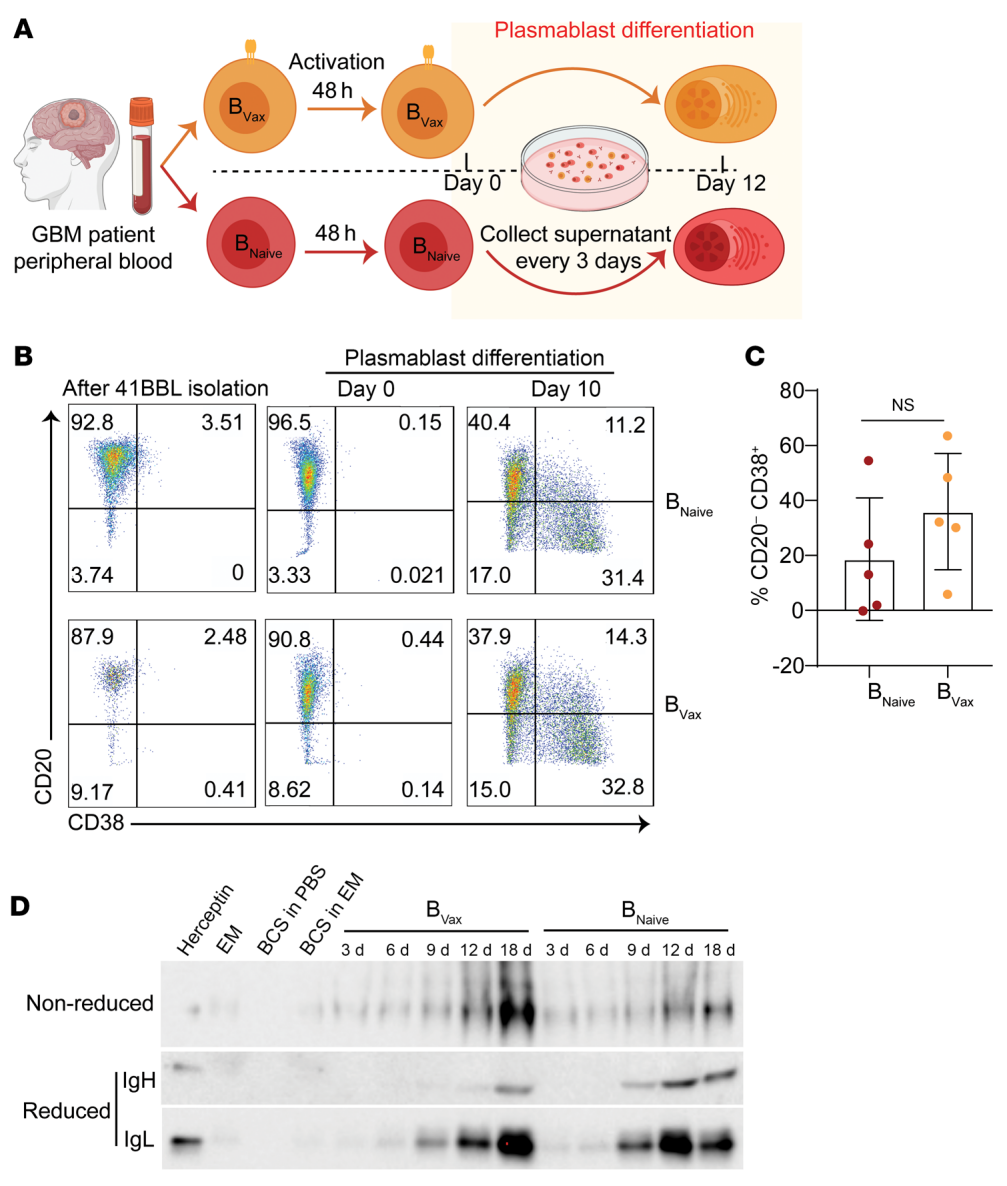
Production of B_Vax_-derived Igs in patients with GBM. (**A**) Schema of the ex vivo generation of GBM patient B_Vax_-derived Abs. (**B**) Dot plots of flow cytometric analysis of CD20 and CD38 expression during different stages in the B_Vax_ activation protocol to generate GBM patient–derived Abs ex vivo. (**C**) Box-and-whisker plot of the percentage of plasmablasts generated at day 10 of B_Vax_/B_Naive_ activation in patients with GBM (*n* = 5 for each group). (**D**) Western blot confirming the presence of Abs in the media during various stages of the ex vivo B_Vax_ activation protocol for patients with GBM. EM, expansion medium; BCS, B cell supplement. Data in **C** are the mean ± SD and were analyzed by 2-tailed Student’s *t* test.

To examine the biological relevance of the murine model, we evaluated the antigenic reactivity of B_Vax_ from patients with GBM. B_Vax_ from patients newly diagnosed with GBM were differentiated into plasmablasts in vitro (Figure 3A). Flow cytometric analysis confirmed that 10 days after activation, approximately 30% of cells generated from either B_Naive_ or B_Vax_ showed a CD19^+^CD20^–^CD38^+^ plasmablast phenotype (Figure 3, B and C), suggesting that both B cell types had a similar in vitro polyclonal potential to differentiate into plasmablasts. Supernatants were then collected every 3 days, and the presence of secreted Igs was confirmed by Western blotting (Figure 3D) and ELISA (Supplemental Figure 4). We detected the production of IgG (both IgH and IgL chains) in both B_Vax_ and B_Naive_ conditions, with clear bands appearing from day 6 onward, confirming the efficacy of the ex vivo generation method in producing GBM patient–derived Abs.

**Figure 4:**
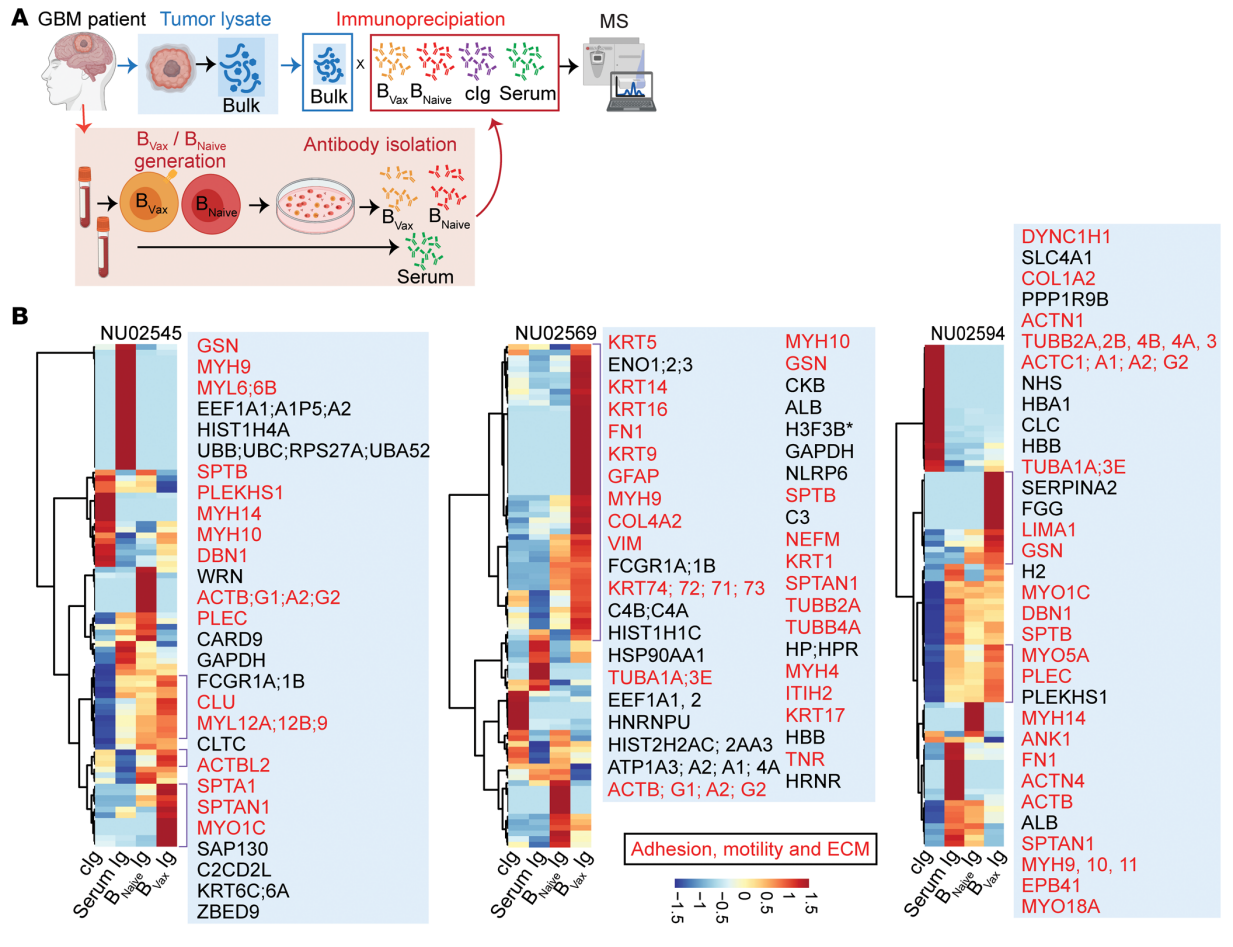
Characterization of B_Vax_-derived Ig reactivity in patients with GBM. (**A**) Schema depicting the protocol for the human IP-MS experiments used to identify tumor-specific antigens recognized by B_Vax_-derived Abs. (**B**) Heatmap revealing hierarchical clustering of GBM tumor antigens recognized by B_Vax_-derived Igs (*n* = 3). Targets related to adhesion, motility, or the ECM are shown in red.

**Figure 5:**
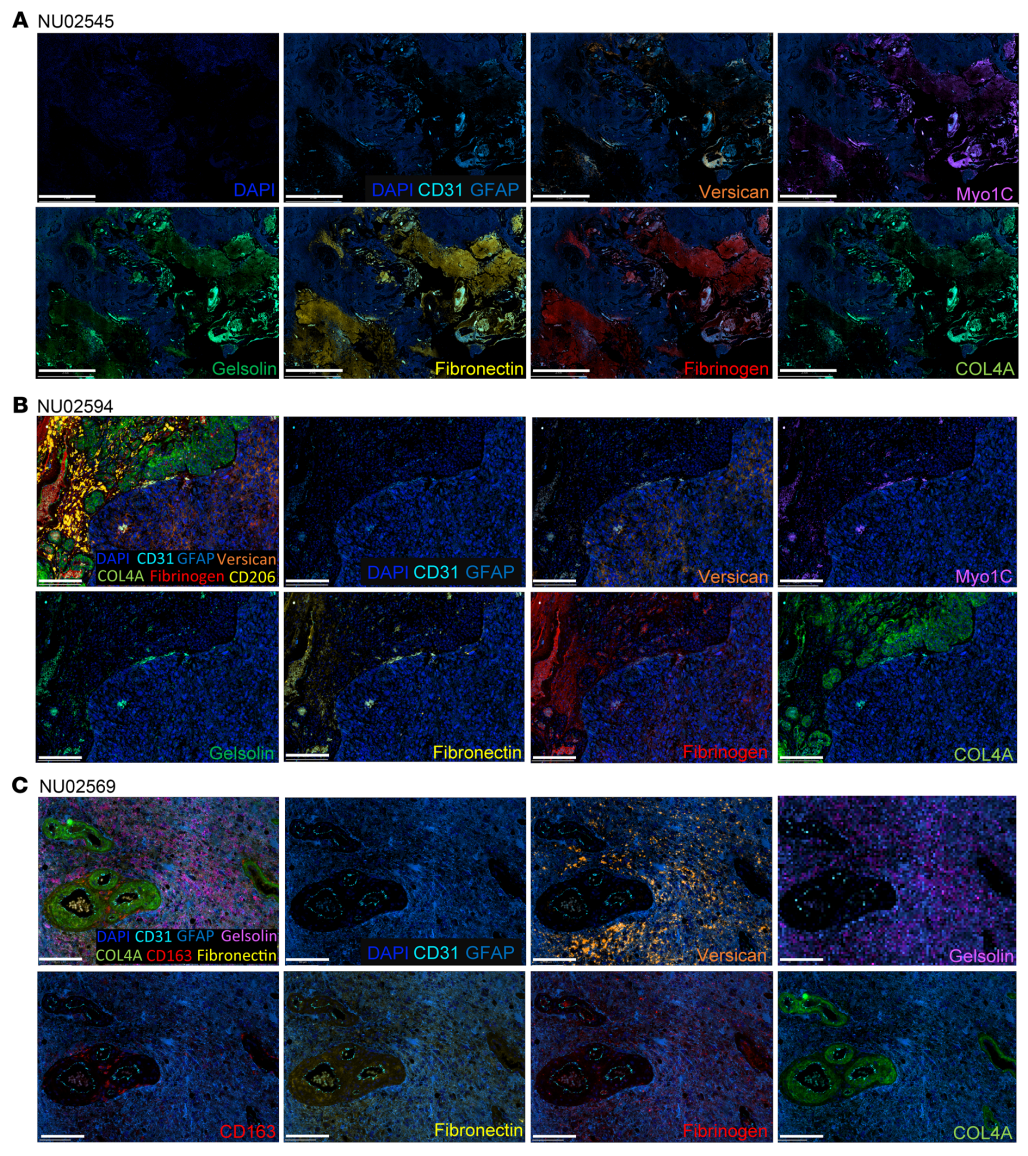
B_Vax_-derived, Ig-recognized antigens are part of ECM. Representative spatial multiplex IF images generated using the COMET system (Lunaphore Technologies) from paired GBM patients (*n* = 3) showing that B_Vax_-derived, Ig-recognized antigens are part of the GBM ECM (including versican, fibronectin, and COL4A), ECM modulators (gelsolin), and proteins involved in cell adhesion and motility (MYO1C and fibrinogen). (**A**) Images for patient NU02545. Scale bars: 2 mm. (**B**) Images for patient NU02594. Scale bars: 200 μm. (**C**) Images for patient NU02569. Scale bars: 200 μm. CD31 (endothelial cells), GFAP (glioma tumor cells and astrocytes), CD163 (macrophage scavenger receptor), CD206 (immunosuppressive macrophages).

In parallel to the murine glioma analysis, Igs from supernatants of the B cell cultures harvested every 3 days were isolated using a Protein A/G Spin Column. Given the limited amount of Igs, the IP-MS was performed using autologous bulk glioma protein homogenates without fractionation (Figure 4A). The patient’s serum Igs were included to evaluate the level of peripheral baseline reactivity to the autologous tumor. The IP-MS data (Figure 4B and Supplemental Table 7) showed heterogeneity across patients (NU02545, NU0569, and NU02594). However, we found several regulators of the ECM (such as fibrinogen, versican core protein, and collagens) and cell motility (such as myosin and actin) across all patients’ B_Vax_ Igs. Furthermore, GO pathway analysis confirmed that B_Vax_ had a preferential reactivity toward biological processes involved in cell migration and motility (Supplemental Figure 5). To corroborate these findings, we conducted additional immunoprecipitation experiments using conditioned medium and cell membrane fractions from GBM43 tumor cells cultured in vitro. These experiments confirmed that B_Vax_ Abs specifically targeted ECM proteins (such as gelsolin, collagens, matrix metalloproteinases, thrombospondins, and laminin) and membrane proteins (such as ILK complex, EGFR, flotillin-2 [FLOT2], and Rho GTPases) enriched in these fractions (Supplemental Figure 6 and Supplemental Table 8), confirming their preferential reactivity. Spatial proteomics and histopathological analysis of tumors from these patients illustrate the spatial distribution of B_Vax_-derived, Ig–recognized antigens within the ECM of GBM (Figure 5). Gelsolin (GSN), fibronectin (FN1), versican (VCAN), fibrinogen (FGB), myosin type 1 C (MYO1C), and collagen type IV α (COL4A) corresponded to perivascular clotting and hemorrhagic areas. The diverse staining patterns across patients NU02545 (Figure 5A and Supplemental Figure 7), NU02594 (Figure 5B, Supplemental Figure 8, and Supplemental Video 1), and NU02569 (Figure 5C, Supplemental Figure 9, and Supplemental Video 2) indicated patient-specific variations in ECM composition and the immune response. Such heterogeneity could explain the inconsistency of B_Vax_-derived Ig reactivity in different patients with GBM. Additionally, we observed B cells in regions near B_Vax_-derived, Ig-recognized antigens (Supplemental Figure 10), and peritumoral brain had low expression of these ECM proteins compared the GBM microenvironment itself (Supplemental Figure 11), indicating therapeutic potential for B_Vax_-derived Igs.

### B_Vax_-Igs inhibit GBM invasion and migration.

**Figure 6:**
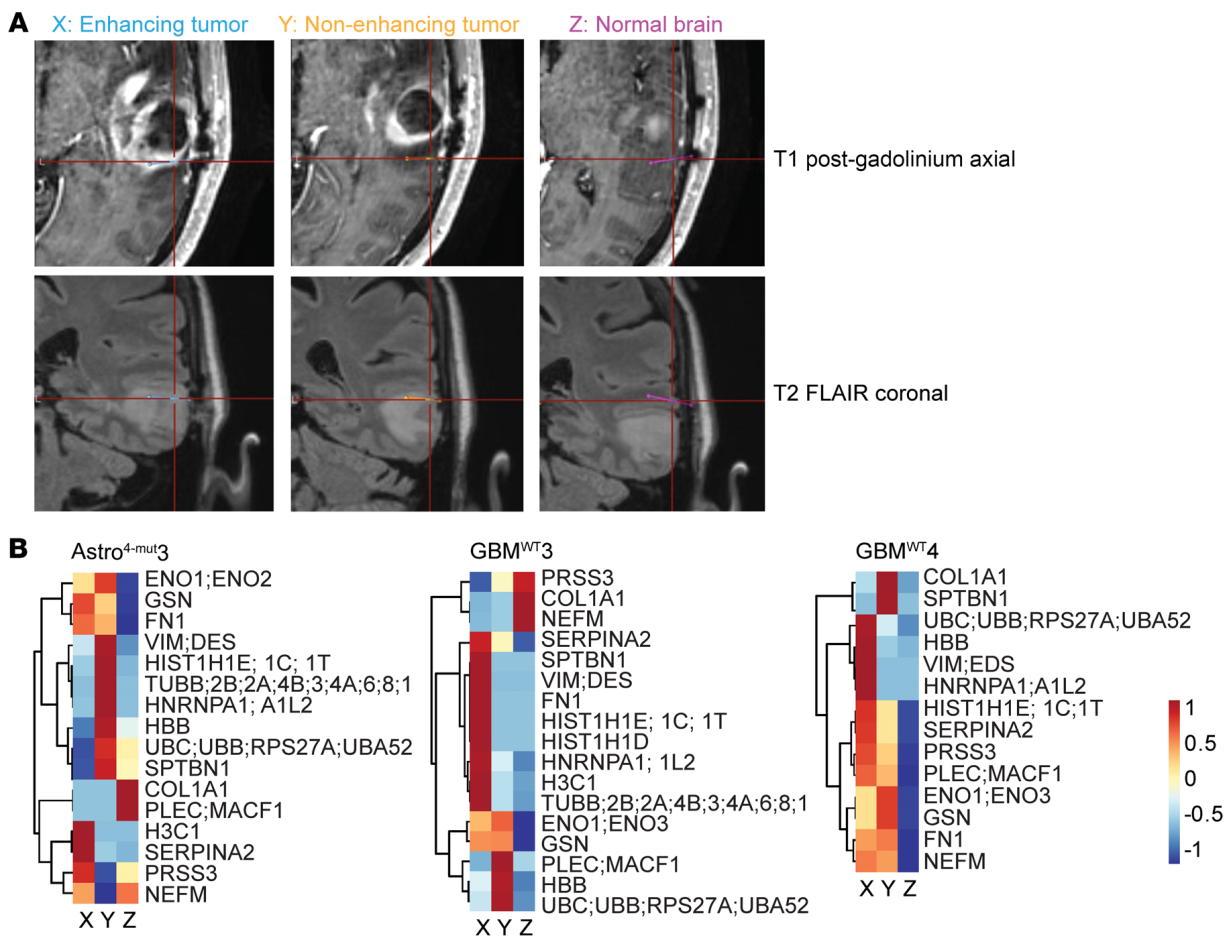
B_Vax_-derived, Ig-recognized antigens are detected in the extracellular fluid with brain microdialysis. (**A**) T1 post-gadolinium axial and T2 fluid-attenuated inversion recovery (FLAIR) coronal MRIs demonstrating the stereotactic target location of each catheter in enhancing tumor, nonenhancing tumor, and normal brain (MRI for patient GBM^WT^3 is shown). (**B**) Heatmap revealed the relative intensity of high-grade glioma antigens recognized by B_Vax_-derived Igs in the micro dialysate. Samples were collected from 3 distinct patients, different from those in Figure 5.

To determine whether patient B_Vax_-derived, Ig-recognized antigens are accessible to the immune system, we performed intraoperative high-molecular-weight microdialysis, which collects proteins secreted into the interstitial fluid space (40), in the tumor and brain tissue adjacent to the tumors of patients with GBM (GBM^WT^3, and GBM^WT^4) and in 1 patient with a grade 4 isocitrate dehydrogenase–mutant (IDH-mutant) astrocytoma (Astro^4-mut^3), followed by MS analysis (Figure 6A). High-molecular-weight catheters (100 kDa) were applied to maximize the volume and diversity of the microdialysis (40). During the resection, catheters were placed in radiographically enhancing tumor (X) and nonenhancing tumor (Y), and in relatively normal brain adjacent to the tumor (Z). MS of the microdialysates revealed that most B_Vax_-derived, Ig-recognized antigens were more abundantly secreted in the enhancing tumors than in normal brains, although 1 patient showed secretion of more B_Vax_-derived, Ig-recognized antigens in the nonenhancing tumors (Figure 6B and Supplemental Table 9). Some B_Vax_-derived, Ig-recognized antigens, such as myosin (MYH) (Figure 4B), were not identified in the microdialysates (Figure 6B). One possible explanation could be that their size surpassed the molecular weight cutoff of the catheters (>100 kDa). Overall, these findings demonstrate the presence of diverse antigenic profiles across heterogeneous zones within each tumor and the potential accessibility of these antigens to the immune system. The presence of these B_Vax_-derived, Ig-recognized antigens in the extracellular fluid reinforces the notion that the tumor microenvironment (TME) is a potential reservoir of significant therapeutic targets.

**Figure 7:**
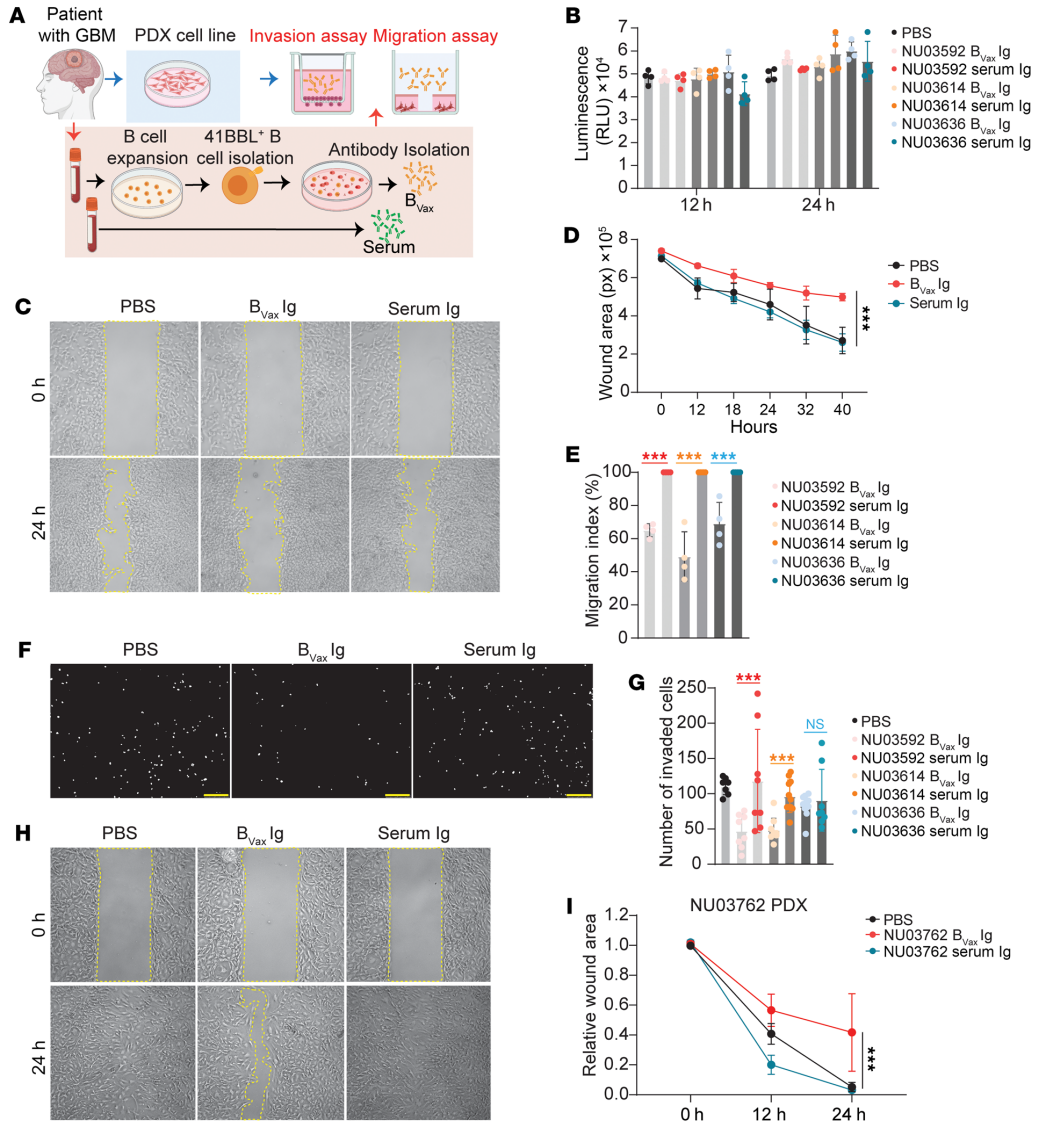
B_Vax_-derived Igs inhibit tumor invasion and migration. (**A**) Schema depicting the protocol for the ex vivo functional assay. (**B**) Cell viability of GBM43 cells after treated with serum- or B_Vax_-derived Igs from patients with GBM (NU03592, NU03614, and NU03636). *n* = 3. (**C**) Representative images of wound areas (marked by yellow lines) on confluent monolayers of GBM43 cells at 0 hours and 24 hours; cells were treated with serum- or B_Vax_-derived Igs from a patient with GBM (NU03592). Original magnification, ×4 (**C**, **F**, and **H**). (**D**) Quantification of the wound area at different time points of GBM43 cells treated with serum- or B_Vax_-derived Igs from a patient with GBM (NU03592). (**E**) Quantification of the migration index of GBM43 cells at 24 hours that were treated with serum- or B_Vax_-derived Igs from patients with GBM (NU03592, NU03614, and NU03636). *n* = 3. (**F**) Representative images and (**G**) quantification of invading GBM43 cells (DAPI^+^) at 24 hours; cells had been treated with serum- or B_Vax_-derived Igs from patients with GBM (NU03592, NU03614, and NU03636). *n* = 3. Each white dot represents a single invaded cell. Scale bars: 250 μm. (**H**) Representative images of wound areas (marked by yellow lines) on confluent monolayers of PDX cells at 0 hours and 24 hours; cells had been treated with serum- or B_Vax_-derived Igs from the same patient (NU03762). (**I**) Quantification of the wound area of PDX cells at different time points; cells had been treated with serum- or B_Vax_-derived Igs from the same patient (NU03762). Data are the mean ± SD. ***P < 0.001, by 1-way ANOVA (**B**, **E**, and **G**) or 2-way ANOVA (**D** and **I**).

The ECM and cell motility are key biological processes controlling tumor cell migration, invasion, and epithelial-mesenchymal transition, which are hallmarks of GBM malignant behavior (41–44). Thus, we hypothesized that B_Vax_ Igs could inhibit ECM and cell motility processes by recognizing these components or regulators. To test this hypothesis, we used a patient-derived xenograft (PDX) cell line (GBM43) and B_Vax_ Igs from 3 patients with GBM (NU03592, NU03614, and NU03636). Igs from autologous plasma samples were used as a control. We performed an ex vivo PDX functional assay including invasion and migration assessment using a commercially available matrix that recapitulates mammalian ECM (Matrigel) (Figure 7A). B_Vax_ Igs did not affect PDX cell viability (assessed by ATP activity, Figure 7B) compared with paired serum Igs. However, B_Vax_ Igs significantly inhibited the PDX cells from migrating (Figure 7, C–E) and invading (Figure 7, F and G). Additionally, we conducted a migration assay using B_Vax_ Igs from patient NU03762 and the corresponding PDX cell line (NU03762 PDX). Consistent with our initial findings, B_Vax_ Igs significantly inhibited migration of the paired PDX cell line (Figure 7, H and I). These results support the potential of B_Vax_ Igs to interfere with key processes involved in GBM progression.

**Figure 8:**
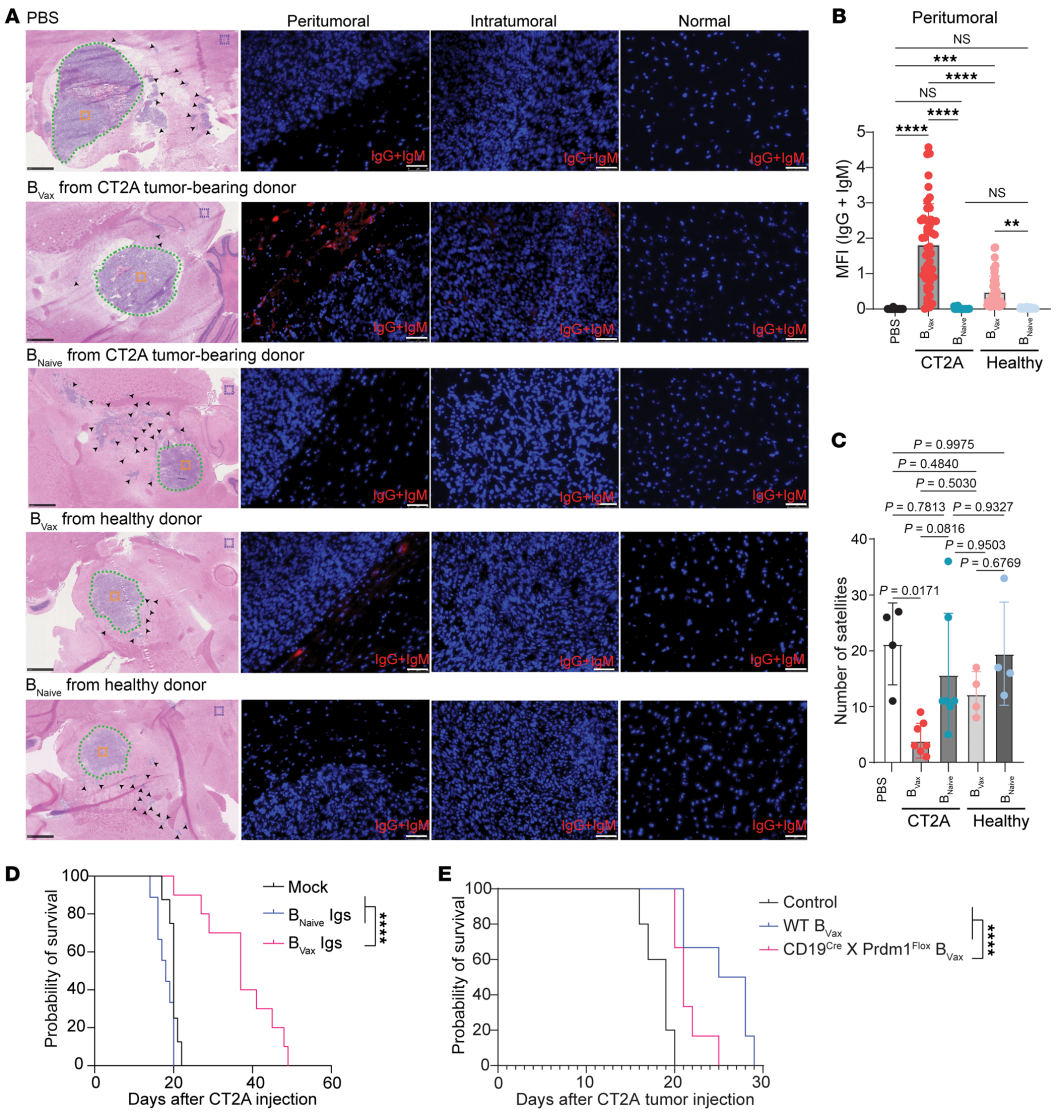
B_Vax_ cells from tumor-bearing mice have a superior ability to produce Abs that localize to the peritumoral region and promote survival of GBM-bearing mice survival. (**A**) Representative images of H&E and IF staining for anti–mouse IgG and IgM to assess the presence and localization of B_Vax_-derived Igs. B_Vax_ or B_Naive_ cells from healthy or CT2A tumor–bearing C57BL/6 mice were adoptively transferred into CT2A tumor–bearing muMT mice. Following treatment, brain tissues were harvested from recipient mice and stained for anti–mouse IgG and IgM (red). H&E-stained images show the organization of the tumors and the locations where the IF images were taken: peritumoral region (dotted green line), intratumoral region (orange box), and relatively normal brain (purple box). (**B**) Quantification of the relative intensity of B_Vax_-Igs in the peritumoral region. A total of 10–15 images were taken around the peritumoral region in each mouse (dotted green line). The MFI of anti–mouse IgG and IgM (red) in each image was quantified using ImageJ as described previously (68, 69). PBS group: *n* = 3; B_Vax_CT2A group: *n* = 5; B_Naive_CT2A group: *n* = 5; B_Vax_healthy group: *n* = 3; B_Naive_healthy group: *n* = 3. Data are representative of 2 independent experiments. (**C**) Quantification of satellites (black arrowhead) away from the CT2A tumor core based on H&E images from each mouse. PBS group: *n* = 3; B_Vax_-CT2A group: *n* = 5; B_Naive_CT2A group: *n* = 5; B_Vax_ healthy group: *n* = 3; B_Naive_healthy group: *n* = 3. The data are representative of 2 independent experiments. (**D**) Survival of CT2A tumor–bearing mice was evaluated in 3 groups: mock-treated (*n* = 8), B_Naive_ Ig–treated (*n* = 9), and B_Vax_ Ig–treated (*n* = 10). (**E**) Survival of CT2A tumor–bearing muMT mice was assessed according to the 3 treatment groups: PBS control (*n* = 5), WT B_Vax_ (*n* = 6), and Prdm1-deficient B_Vax_ (*n* = 6). Data are presented as the mean ± SD. **P < 0.01, ***P < 0.001, and ****P < 0.0001, by 1-way ANOVA (**B** and **C**) or log-rank test (**D** and **E**).

To provide direct evidence of B_Vax_-induced Igs in tumors, we conducted additional experiments in which B_Vax_ cells from healthy or CT2A tumor–bearing C57BL/6 mice were adoptively transferred into CT2A tumor–bearing muMT (B cell–KO) mice. Following treatment, we harvested brain tissues from recipient mice and performed immunofluorescence (IF) staining for anti–mouse IgG and IgM to assess the presence and localization of B_Vax_-derived Abs. Our results demonstrated that mice receiving B_Vax_ cells from CT2A tumor–bearing donors had significant IgG and IgM staining in the peritumoral region compared with those treated with B_Vax_ cells derived from healthy donors or with B_Naive_ cells (Figure 8, A and B). Similar results were observed in the GL261 model, in which B_Vax_ or B_Naive_ cells from GL261 tumor–bearing C57BL/6 mice were adoptively transferred into GL261 tumor–bearing muMT (B cell–KO) mice (Supplemental Figure 12). Histological analysis of muMT mice treated with B_Vax_ cells from CT2A tumor–bearing donors showed a decrease in satellite formation away from the tumor core, indicating the disruption of cell invasion (Figure 8, A and C). Consistent with our previously reported results (13), only mice with orthotopically implanted CT2A tumors treated with B_Vax_ Igs had a significant increase in median survival (Figure 8D). To further assess the importance of the role of B_Vax_ Igs, we conducted experiments in which we treated CT2A tumor–bearing mice with B_Vax_ generated from WT mice or mice with B cells deficient in Prdm1 (*Cd19^Cre^ Prdm1^fl^* mice, provided by Nicole Baumgarth, Johns Hopkins Medicine, Baltimore, Maryland, USA). Prdm1 encodes Blimp1, a key factor for the development of Ig-secreting plasma cells (45, 46). Notably, the survival of Prdm1-deficient, B_Vax_-treated mice was further reduced (Figure 8E), underscoring the essential role of B_Vax_-induced Igs in mediating the survival benefits observed with B_Vax_ treatment.

On the basis of these results, we conclude that B_Vax_ has the potential to produce Abs reactive to the tumor ECM and components of the cell motility and could interfere with the ability of the tumor to migrate and invade nontumor tissue and to ultimately affect overall tumor growth.

## Discussion

Here, we show that B_Vax_ elicited antitumor reactivity, as evidenced by selective migration to glioma-bearing brains, differentiation into plasmablasts, and secretion of specific Igs. B_Vax_-derived Igs bind to factors predominantly involved in cell motility and the ECM, essential for GBM invasion and motility (42, 47–49). These Abs are also functionally active against the key processes of cancer progression, revealing a strategy for developing novel immunotherapeutic strategies against GBM.

The B_Vax_-derived Ig recognition of specific ECM components, such as gelsolin, fibronectin, fibrinogen, versican, and collagens, is particularly intriguing. Traditionally seen as a physical scaffold for cells, the ECM is now increasingly recognized for its role in modulating tumor behavior, progression, and response to therapy (43, 44). The ECM and the hypoxic microenvironment orchestrate the mesenchymal transition, a biological process associated with the aggressive pathological properties of GBM and therapeutic resistance (49–51). Recently, the finding of collagen 1 α1–abundant (COL1A1-abundant) oncostreams (52) and cancer-associated fibroblasts (53) in GBM and their protumor effects reinforce the concept that components of the ECM might not be mere bystanders but could actively participate in the progression of GBM. The preferential reactivity of B_Vax_ Abs observed in murine models and human GBM samples highlights its translational potential.

To date, relatively few antigens recognized by TIB cell–derived Abs have been identified in other cancer models, partially limited by the low TIB numbers from fresh tumors (54–60). Here, by expanding and differentiating B_Vax_ into Ab-secreting plasmablasts, we were able to identify potential antigens recognized by B_Vax_-derived Abs in mouse and human GBM models. This method can also be used in other cancer models, expanding the repertoire of TIB target antigens.

Most TIB target antigens identified in patient tissue samples span across the nuclear, cytoplasmic, and extracellular compartments (24). Our data demonstrated that cytoplasmic and extracellular proteins could both be recognized by B_Vax_ Abs. While it is expected that B cell–derived Igs could recognize extracellular proteins, the detection of intracellular cytoplasmic proteins was surprising. However, the analysis of the GBM secretome obtained from microdialysis of enhancing tumor, nonenhancing tumor, and normal brain showed that cytoplasmic proteins can be detected in the extracellular fluid, hinting that cytoplasmic proteins are also accessible to the immune system. However, the mechanism behind this is still not clear. One could hypothesize that soluble antigens from the interstitial fluid could travel to regional lymph nodes, such as the deep cervical lymph nodes (61), and potentially activate B cells.

Although our data indicate that B_Vax_ Igs contributed significantly to improved survival, it is essential to consider the potential contributions of other immune mechanisms of B_Vax_, such as CD8^+^ T cell activation, which also plays an important role in the B_Vax_-conferred therapeutic effect (13, 62). Further studies are needed to delineate the interplay between B_Vax_ Igs and other immune mechanisms of B_Vax_ and how these mechanisms collectively enhance patient outcomes.

The heterogeneity observed in the B_Vax_ antigenic reactivity across different patients with GBM reminds us of the complex and diverse nature of GBM tumors. Although certain regulators like fibrinogen, myosin, and collagens emerged consistently, future research should aim at identifying and characterizing other potential tumor antigens bound by B_Vax_ Igs using a larger cohort of patient samples. In conclusion, our research highlights the therapeutic potential of B_Vax_-derived Igs in GBM therapy. Through their unique tumor-reactive nature, B_Vax_ and the Abs they produce offer a promising strategy against this formidable malignancy. Future studies should focus on how to unleash the full potential of B_Vax_ in an immune-suppressive GBM TME.

## Methods

### Sex as a biological variable.

In this study, both male and female mice were used in the animal model experiments to investigate the effects of B cell–based therapy on GBM growth. However, sex was not considered as a biological variable in any of the analyses. For studies involving human samples, sex was also not considered as a biological variable because of the limitations in sample availability and the specific focus of the study.

### Human specimens.

The Nervous System Tumor Bank collected all human tissue samples at Northwestern University and involved 9 patients with IDH-WT GBM. The Mayo Clinic Cancer Center collected all human tissue samples at the Mayo Clinic. Samples collected from patients with high-grade glioma included freshly resected tumors, peripheral blood, frozen tumors, and paraffin-embedded tissue sections. A neuropathologist reviewed all H&E sections to confirm that the sample included the presence of least 50% tumor cells based on cellularity.

### Cell lines.

CT2A cells were obtained from MilliporeSigma, GL261 cells were obtained from the National Cancer Institute (NCI), NIH, and GBM43 PDX glioma cell lines were obtained from David James (Northwestern University, Chicago, Illinois, USA). NU03762 PDX cells were obtained from Craig M. Horbinski (Northwestern University). NU03762 PDX cells were maintained in complete RPMI, consisting of RPMI supplemented with 10% FBS (Hyclone), 100 U/mL penicillin (Corning), 100 mg/mL streptomycin (Corning), 0.1% 2-mercaptoethanol (MilliporeSigma), 2 mM l-glutamine (Invitrogen, Thermo Fisher Scientific), 25 mM HEPES (Invitrogen), and 1 mM sodium pyruvate (Invitrogen, Thermo Fisher Scientific). The other cell lines (CT2A, GL261, GBM43) were maintained in DMEM (Corning) supplemented with 10% FBS (Hyclone), 100 U/mL penicillin (Corning), and 100 mg/mL streptomycin (Corning) and incubated at 37°C in 5% CO_2_. Every 2 months, the cell lines were tested for mycoplasma contamination using the Universal Mycoplasma Detection Kit (American Type Culture Collection [ATCC], 30-1012K).

### Murine models.

Mice used in this study included C57BL/6 and muMT mice purchased from The Jackson Laboratory and bred for use in the experiments. *Cd19^Cre^* *Prdm1^fl^* mice were provided by Nicole Baumgarth (Johns Hopkins Medicine, Baltimore, Maryland, USA). Animal experiments were initiated when the mice were 6–8 weeks old. All animals were housed at the Simpson Querry Center for Comparative Medicine in a dedicated pathogen-free animal facility with 12-hour light/12-hour dark cycles and ad libitum access to food and water. For in vivo studies, the sample size for each experiment is indicated in the figure legend. The investigators were not blinded to the groups for any experiments. This study incorporated sex as a biological variable by including both male and female mice.

### Intracranial tumor implantation.

Each mouse was implanted with 1 × 10^5^ glioma cells in a total volume of 2.5 μL PBS. Mice were anesthetized with ketamine (100 mg/kg) and xylazine (10 mg/kg) via intraperitoneal injection. After shaving the surgical site and disinfecting with povidone-iodine and 70% ethanol, an incision was made at the midline to access the skull. A 1 mm diameter burr hole was drilled 2 mm posterior to the coronal suture and 2 mm lateral to the sagittal suture. Injections were performed using a Hamilton syringe fitted with a 26 gauge blunt needle at a depth of 3.5 mm. The injection site was then sutured closed.

### B_Vax_/B_Naive_ generation.

Cells were generated as described previously (13, 14). Briefly, 4-1BBL^+^ B cells were isolated and activated with anti-CD40 Ab (CD40 agonism), supplemented with BAFF (B cell survival factor), and stimulated with IFN-γ in complete RPMI media (cRPMI) (RPMI 1640 supplemented with 10% FBS, sodium pyruvate, MEM amino acids, HEPES, 2-mercaptoethanol, and penicillin/streptomycin) to generate B_Vax_. The 4-1BBL^–^ B cells were cultured with BAFF in cRPMI to generate B_Naive_.

### GBM patient–derived B_Vax_/B_Naive_ Ig generation.

B_Vax_/B_Naive_ cells were cultured using the ImmunoCult Human B Cell Expansion Kit (100-0645, STEMCELL Technologies). Cells were seeded at a density of 5 × 10^5^ cells/mL in B cell expansion medium and incubated at 37°C in a CO_2_ incubator. Every 3 days, supernatants were harvested and quantified for IgG using the IgG (total) Human Uncoated ELISA Kit (88-50550-22, Invitrogen). The supernatants were then stored at –80°C, and cells were replenished with fresh expansion medium. After several rounds of supernatant collection, all supernatants were pooled, and the Igs were purified using a NAb Protein A/G Spin Column (89962, Thermo Fisher Scientific).

### Murine B_Vax_/B_Naive_ Ig generation.

The generation of murine Igs is depicted in Figure 2A. Briefly, B_Vax_/B_Naive_ cells were adoptively transferred into muMT mice. After 2 weeks, blood from B_Vax_/B_Naive_ recipient mice was collected, and Igs were isolated using a Protein A/G Spin Column.

### GBM patient–derived tumor lysate for IP-MS.

Fresh tumors and paired blood samples from patients NU02545, NU02569, and NU02594 were collected by the Northwestern Brain Tumor Bank. Tumors were homogenized in NP40 buffer (40 mM HEPES, 120 mM NaCl, 1 mM EDTA, 10 mM NaPP, 50 mM NaF, 0.5% NP40, and 10 mM β-glycerophosphate) to which protease and phosphatase inhibitors were added (MilliporeSigma). Protein quantification was performed using a Bradford Assay (Bio-Rad, catalog 500-0006) according to the manufacturer’s guidelines.

### Murine GBM tumor lysate for IP-MS using cellular fractionation.

CT2A cells were implanted intracranially into C57BL/6 mice. After 21 days, the brains were collected, and the tumors were dissected and flash-frozen. As per the manufacturer’s guidelines, cellular fractionation was performed with the Thermo Fisher Mem-PER Plus Kit (catalog 89842Y). Protein quantification was performed using a Bradford Assay (Bio-Rad, catalog 500-0006) according to the manufacturer’s guidelines.

### Immunoprecipitation.

Cell/tissue lysates were incubated and tumbled overnight at 4°C with either B_Vax_-derived Igs or IgG control. B_Vax_-derived Igs were generated in vivo for the murine samples and ex vivo for patient-derived samples as described in Figure 2A and Figure 3A. Pulldown experiments were then performed with magnetic Dynabeads Protein G (Thermo Fisher Scientific). For GBM patient–derived samples, 1–2 mg tumor lysate was used with 20–60 μg Igs. For murine intracranial GBM tumor samples, 1.5 mg tumor lysate was used with 30 μg Igs. For GBM43 cell–derived samples, 2–4 mg membrane or conditioned medium proteins were used with 40–120 μg Igs. For CT2A cell–derived samples, 8 mg membrane or conditioned medium proteins were used with 160 μg Igs. Bead-protein complexes were isolated and then washed 3 times with lysis buffer and 2 times with Triton X buffer (40 mM HEPES, 120 mM NaCl, 1 mM EDTA, 10 mM NaPP, 50 mM NaF, 0.5% Triton X, and 10 mM β-glycerophosphate). The beads were then boiled for 10 minutes at 95°C in 2X Laemmli sample buffer, 20% of each sample was resolved by SDS-PAGE, and 80% was submitted to the proteomics core for MS analysis.

### Proteomics immunoprecipitation analysis using LC-MS/MS.

Liquid chromatography tandem MS (LC-MS/MS) was performed as previously described (63). For visualization of protein targets identified through IP followed by MS, R programming language was used, leveraging specialized packages such as “ggplot2” and “pheatmap.” Our datasets encompassed protein measurements derived from 3 tumor samples from patients with GBM, in vitro cultures, and mouse samples, which included protein targets extracted from control Ig, B_Naive_ or serum Ig, and B_Vax_ Ig groups. For heatmap visualization, the Ward’s method (64) was adopted to cluster samples, ensuring a more coherent and intuitive presentation.

### Flow cytometry.

Flow cytometry was performed as described previously (13). The following anti-human Abs were used (all from BioLegend): 4-1BBL PerCP-Cy5.5 (5F4) (catalog 311518), CD19 Pacific blue (HIB19) (catalog 302224), CD20 BV510 (2H7) (catalog 302340), and CD38 Alexa Fluor 700 (HB-7) (catalog 356623). All samples were analyzed on a BD Symphony flow cytometry analyzer with proper single-color controls and compensation. All final analysis and data output were performed using FlowJo software (BD).

### Western blot analysis.

B_Vax_ was generated ex vivo from peripheral blood samples from patients with GBM and subsequently activated as described previously (13). Supernatants were collected every few days during the activation protocol and prepared for Western blotting in nonreduced and reduced fractions. Reduced samples were prepared using 4× Laemmli Sample Buffer (Bio-Rad). Equal volumes of supernatant were loaded onto a gel. Supernatants were used as the primary Ab. The secondary Ab used was anti–human IgG-peroxidase (MilliporeSigma, A0293).

### BCR sequencing.

B_Vax_ and B_Naive_ were generated as previously described (14). Then, cells were washed twice with PBS. TIB cells were magnetically isolated using CD19-biotin (clone 6D5, BioLegend, 115504) and anti-biotin Microbeads (Miltenyi Biotec, 130-090-485). RNA isolation from B_Vax_, B_Naive,_ and TIB cells was performed using TRIzol (Invitrogen). BCR sequencing and bioinformatics analysis were performed by Adaptive Biotechnologies using the ImmunoSEQ platform.

### Sequential IF multiplex staining.

The multiplex panel included the following unconjugated Abs: CD31 (endothelial cells; Abcam, EPR3131), GFAP (glioma tumor cells and astrocytes; Abcam, EPR1034Y), CD163 (macrophage scavenger receptor; Abcam, EPR19518), CD206 (immunosuppressive macrophages; Abcam, ab64693), COL4A (collagen 4 subtypes A1/A2; Abcam, ab6586), versican (MilliporeSigma, HPA004726), fibrinogen (Abcam, ab34269), fibronectin (Abcam, ab2413), gelsolin (Cell Signaling Technology, D9W8Y), MYO1C (MilliporeSigma, HPA001768), and CD20 (Dako Agilent Technologies, L26). All Abs were validated using conventional IHC and/or IF staining in conjunction with the corresponding fluorophore and the spectral DAPI (Thermo Fisher Scientific, 62247) counterstain. For optimal concentration and the best signal/noise ratio, all Abs were tested at 3 different dilutions, starting with the manufacturer-recommended dilution (MRD), then MRD/2 and MRD/4. Secondary Alexa Fluor 555 (Thermo Fisher Scientific, A32727) and Alexa Fluor 647 (Thermo Fisher Scientific, A32733) were used at 1:200 and 1:400 dilutions, respectively. The optimizations and full runs of the multiplex panel were executed using the sequential IF (seqIF) methodology integrated into the Lunaphore COMET platform (characterization 2 and 3 protocols, and seqIF protocols, respectively; ref. 65). Staining was done on a maximum of 4 tissue slides simultaneously, where automated cycles of 2 Abs were stained at a time, followed by fully automated imaging and elution, with no sample manipulation required. All reagents were diluted in Multistaining Buffer (BU06, Lunaphore Technologies). The elution step lasted 2 minutes for each cycle and was performed with Elution Buffer (BU07-L, Lunaphore Technologies) at 37°C. Quenching lasted 30 seconds and was performed with Quenching Buffer (BU08-L, Lunaphore Technologies). Staining incubation durations were 4 minutes for all primary Abs and 2 minutes for secondary Abs. Imaging was performed in Imaging Buffer (BU09, Lunaphore Technologies) with an integrated epifluorescence microscope at ×20 magnification. Image registration was performed immediately after conclusion of the staining and imaging procedures by COMET Control Software. Each seqIF protocol resulted in a multilayer OME-TIFF file, in which the imaging outputs from each cycle were stitched and aligned. COMET OME-TIFF files contained a DAPI image, intrinsic tissue autofluorescence in TRITC and Cy5 channels, and a single fluorescent layer per marker. Markers were subsequently pseudocolored for visualization of markers in the Viewer from Lunaphore.

### Intraoperative microdialysis.

Intraoperative microdialysis was performed at Mayo Clinic during 3 standard-of-care glioma resections based on previously published methods (40), including 1 for a recurrent grade 4 IDH-mutant astrocytoma (Astro^4-mut^3), 1 primary GBM (GBM^WT^3), and 1 recurrent GBM (GBM^WT^4). Briefly, 3 high-molecular-weight microdialysis catheters (100 kDa; M Dialysis 71 High cutoff brain microdialysis catheters) were inserted into radiographically diverse regions (enhancing, nonenhancing, and normal brain) based on stereotactic neuronavigation. Microdialysis was performed at a flow rate of 2 μL/min using the 107 microdialysis pump and perfusion fluid with 3% dextran 500 kDa to improve analyte recovery. Catheters were flushed prior to insertion to minimize dead space, and microvials were then changed every 20 minutes until the sampling area needed to be resected. Microdialysates were split into 2 aliquots and then placed on dry ice. The third aliquot after catheter insertion was sent for MS proteomics analysis at the Mayo Clinic Proteomics Core via LC-MS/MS for label-free relative quantitation by intensity-based abundance quantity (iBAQ).

### Migration assay.

This assay used a silicone insert with a defined cell-free gap (80206, IBIDI), as described previously (66). Briefly, cells were seeded at 3.5 × 10^4^ per well of culture insert chamber (80206, IBIDI). Cells were left undisturbed for approximately 12 hours in a 37°C CO_2_ incubator. After successful attachment, cells were then washed with Dulbecco’s PBS (DPBS), and the silicone insert was carefully lifted using sterile forceps. Culture medium was switched to cDMEM containing 100 μg/mL B_Vax_ or serum Abs. At different time points, imaging was obtained by bright-field via Leica Microscope until the visible gap closed. The distance or area between cells was measured via pixels on Fiji ImageJ. Migration index was calculated as follows: migration index (%) = (wound area at 0 hours – wound area at 24 hours)/wound area at 0 hours × 100.

### Invasion assay.

For the invasion assay, a Corning’s Matrigel Invasion Chamber (354480, Corning) was used. Briefly, after rehydration of the chamber, 5 × 10^4^ cells were suspended in DMEM and 100 μg/mL B_Vax_ or serum Abs and seeded into the invasion chamber. The bottom chambers were filled with 750 μL cDMEM, which contained DMEM, 10% FBS, 100 U/mL penicillin, and 100 mg/mL streptomycin. After a 24-hour incubation, invaded cells were fixed overnight in 4% formaldehyde and then washed and stained with DAPI (P36931, Thermo Fisher Scientific) as described previously (67). The membrane was imaged, and invading cells were counted using Fiji ImageJ.

### Cell viability assay.

The CellTiter-Glo 2.0 Cell Viability Assay kit (G9242, Promega) was used to assess viability of PDX cells after treatment with B_Vax_-derived Abs or serum Abs (100 μg/mL), as described by the manufacturer.

### Direct evidence of B_Vax_-induced Igs in tumors.

In the CT2A glioma model, B_Vax_ or B_Naive_ cells from healthy or CT2A tumor–bearing C57BL/6 mice were adoptively transferred into CT2A tumor–bearing muMT mice 7 days after tumor injection. Each mouse received 2 × 10^6^ cells every 3 days. Seven days after the third treatment, brain tissues were harvested from recipient mice, frozen with OCT and sectioned, and then stained for anti–mouse IgG and IgM (Cy3 AffiniPure Goat Anti–mouse IgG + IgM [H+L], 1:500, Jackson ImmunoResearch, 115-165-044). Because muMT mice do not have endogenous Igs, using anti–mouse Ig will detect only B_Vax_- or B_Naive_-derived Abs, allowing for specific identification of these Abs. Nearby sections were stained for H&E to check the organization of the tumors. IF images were taken from the peritumoral region, the intratumoral region, and relatively normal brain. For quantification of Ig intensity, 10–15 images were taken around the peritumoral region in each mouse. The MFI of anti–mouse IgG and IgM (red) in each image was quantified as previously described using ImageJ (NIH) (68, 69). To evaluate the invasive feature of CT2A tumor cells after treatment, satellites away from the CT2A tumor core were quantified on the basis of H&E images from each mouse (70). In GL261 glioma model, B_Vax_ or B_Naive_ cells from GL261 tumor–bearing C57BL/6 mice were adoptively transferred into GL261 tumor–bearing muMT mice 7 days after tumor injection. Each mouse received 2 × 10^6^ cells every 3 days. Seven days after the second treatment, brain tissues from recipient mice were harvested, stained and quantified as in the CT2A glioma model.

### Essential role of B_Vax_ induced Igs.

CT2A tumor–bearing, B cell–deficient mice were treated with purified B_Vax_ or B_Naive_ Igs (12.5 μg/mouse/injection) as described previously (13), and their survival was monitored. Additionally, CT2A tumor–bearing, B cell–deficient mice were treated with10^6^ B_Vax_ generated from WT or *Cd19^Cre^ Prdm1^fl^* mice (B cells deficient in Prdm1 were provided by Nicole Baumgarth, Johns Hopkins Medicine, Baltimore, Maryland, USA), and their survival was monitored.

### Statistics.

GraphPad Prism version 8 (GraphPad Software) and R version 4.2.3 (R Foundation for Statistical Computing) were used for all statistical analyses. The sample size for the experiments was 3 or more. Results are represented as the mean ± SD unless otherwise indicated. Comparisons between 2 groups were conducted using a 2-tailed Student’s *t* test. Comparisons between more than 2 groups were conducted using a 1-way ANOVA with Tukey’s or Dunnett’s post hoc multiple-comparison test. For animal survival experiments, Kaplan-Meier survival curves were generated, and a log-rank test was applied to compare survival distributions. All reported *P* values are 2 sided and were considered statistically significant at a *P* value of less than 0.05.

### Study approval.

All animal experiments conducted in this study were reviewed and approved by the IACUC of Northwestern University under protocol number ISO16696. The study protocols adhered to the IACUC’s guidelines to ensure the ethical treatment of animals. For studies involving human samples, the research was reviewed and approved by the IRB of Northwestern University (protocol no. STU00202003) and the Mayo Clinic (protocol no. 19-004694). All patients who contributed to this study signed a written consent form, and the study was conducted according to the US Common Rule of Ethical Standards.

### Data availability.

The mass spectrometric proteomics data were deposited in the ProteomeXchange Consortium database via the PRIDE (71) partner repository and are available via ProteomeXchange (accession code PXD046712; project webpage: http://www.ebi.ac.uk/pride/archive/projects/PXD046712; FTP download: https://ftp.pride.ebi.ac.uk/pride/data/archive/2024/09/PXD046712). Additionally, the supporting data values for all figures and analyses are provided in the Supporting Data Values file, which is included with the online supplemental material.

## Author contributions

CLC conceived the project. BAC, SW, MF, and CLC designed the study. SW, BAC, JLK, HN, VA, GVC, HW, IEO, DH, VA, MD, LKB, TYC, CW, AR, PZ, LCP, KM, CRC, TB, MF, and CLC were involved in data acquisition. BAC, SW, and CLC performed the statistical analysis. SW, BAC, HN, JM, MSL, AUA, CRC, TB, CMH, RS, ABH, AMS, MF, and CLC were involved in the interpretation of data. AR was responsible for the mouse colonies. BAC helped with animal surgery. SW, BAC, and CLC prepared the manuscript.

## Supplementary Material

Supplemental data

Supplemental table 1

Supplemental table 2

Supplemental table 3

Supplemental table 4

Supplemental table 5

Supplemental table 6

Supplemental table 7

Supplemental table 8

Supplemental table 9

Supplemental video 1

Supplemental video 2

Supporting data values

## Version 1 08/29/2024

In-Press Preview

## Version 2 10/15/2024

Electronic publication
