# Supplementary material for: B cell–based therapy produces antibodies that inhibit glioblastoma growth
Source: J Clin Invest. 2024 Aug 29;134(20):e177384. doi: 10.1172/JCI177384 (PMC11473152; doi:10.1172/JCI177384)
Supplement: Supplemental data [file jci-134-177384-s182.pdf]

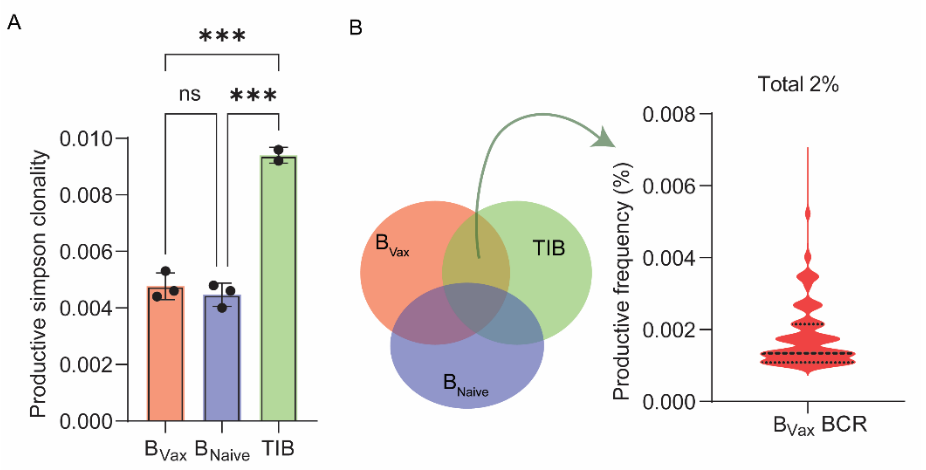

Supplementary Figure 1. Quantitative analysis of BCR-seq data. (A) Relative diversity of B_{Vax} and B_{Naive} BCR repertoires. Values closer to 0 indicate a more diverse repertoire, while values closer to 1 indicate a more clonal population that is dominated by one or a few highly-abundance clones. **(B)** Portion of the B_{Vax} BCRs that overlap with glioma-infiltrating B cells but absent in B_{Naive} BCRs.

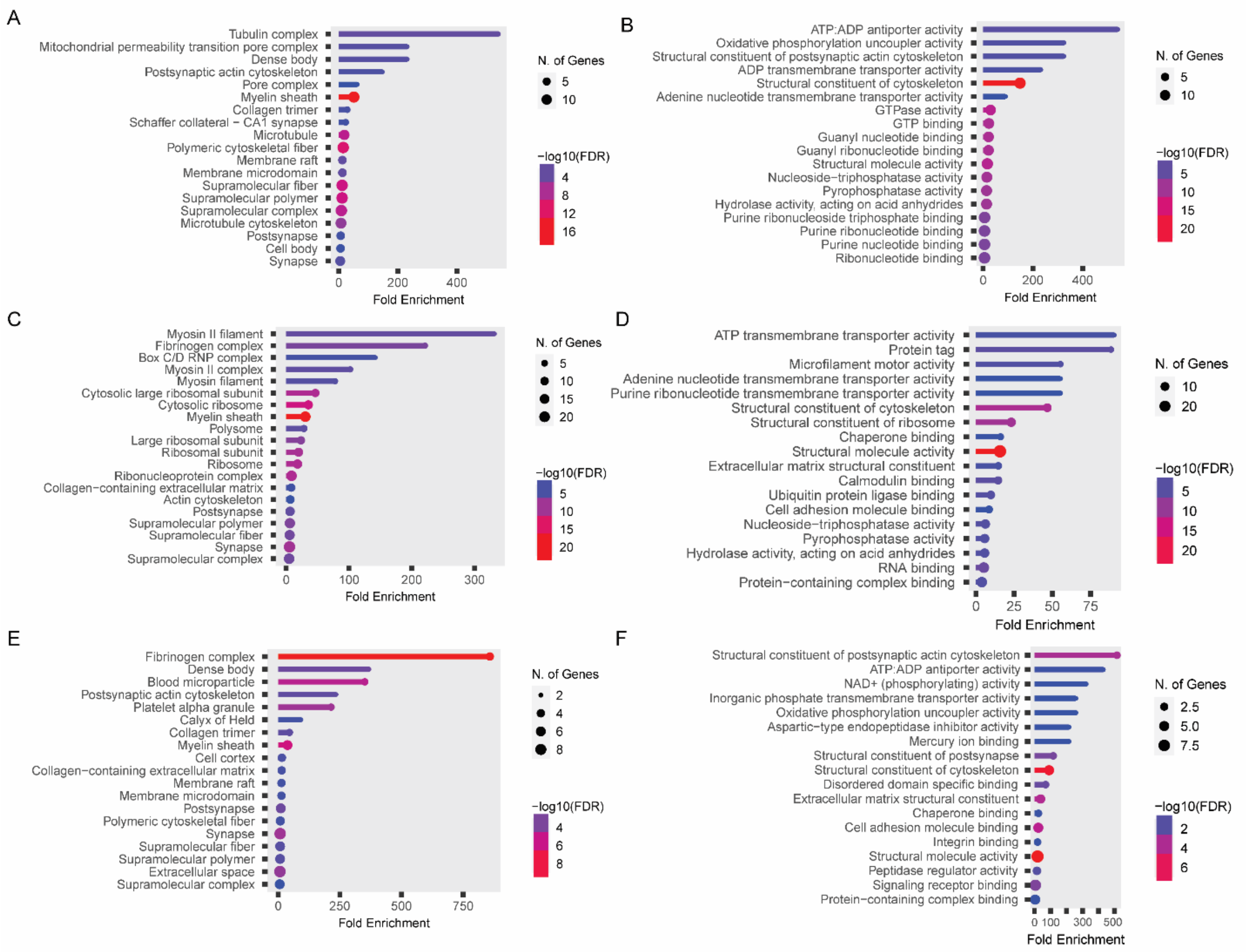

Supplementary Figure 2. Gene Ontology (GO) analysis of proteins recognized by mouse B$_{Vax}$ produced Igs. False discovery rate (FDR) of GO analysis was acquired from ShinyGO 0.77. p <0.05. (**A, C** and **E**) Top 20 GO cellular component terms from 3 replicates (Triplicate 1, Triplicate 2 and Triplicate 3 respectively). (**B, D** and **F**) Top 20 GO molecular function terms from 3 replicates (Triplicate 1, Triplicate 2 and Triplicate 3 respectively).

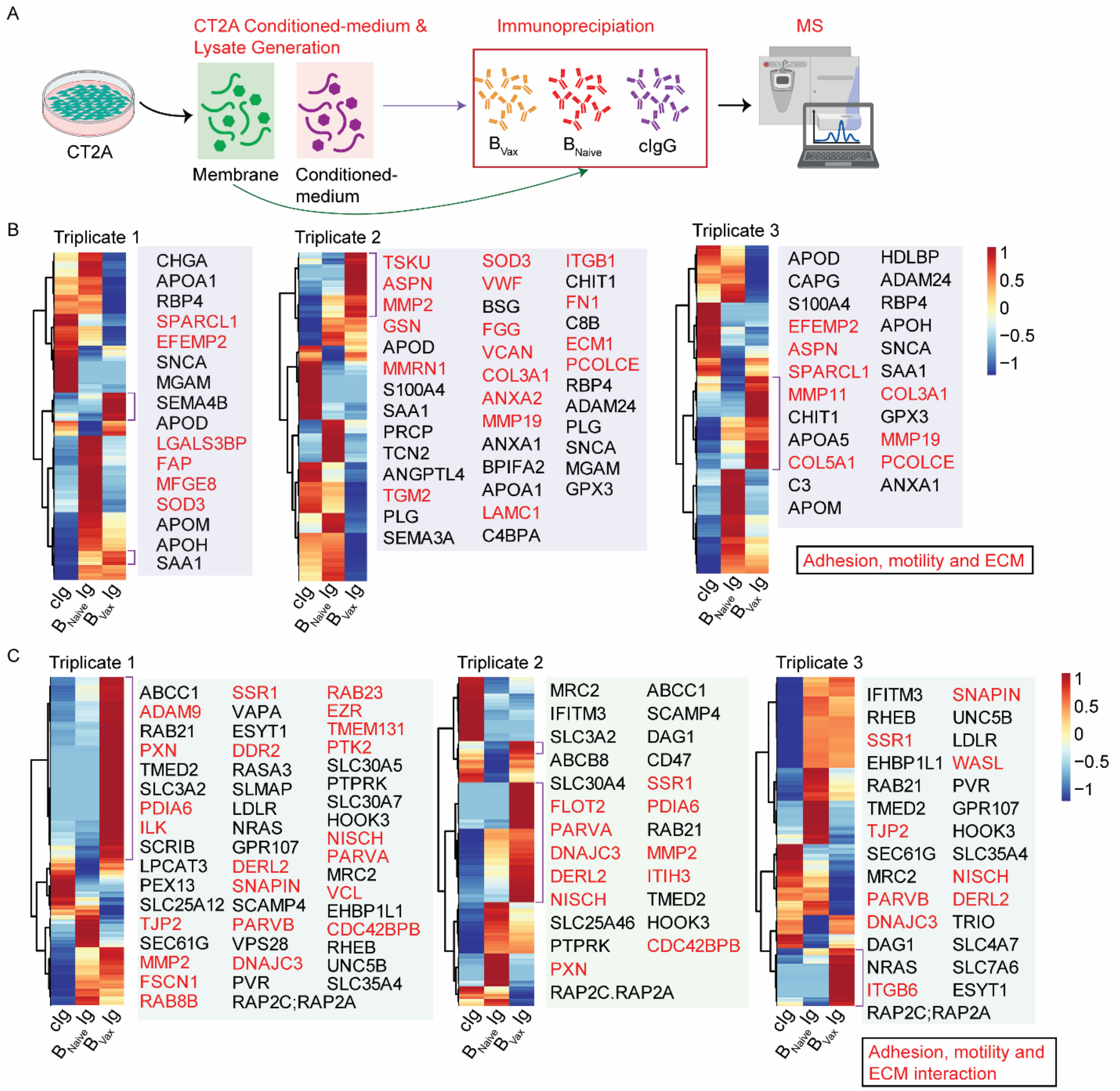

Supplementary Figure 3. Characterization of murine B_{Vax}-derived Ig reactivity. (**A**) Schema depicting the protocol for the murine immunoprecipitation-mass spectrometry (IP-MS) experiments used to identify tumor-specific antigens recognized by B_{Vax}-derived Igs from CT2A tumor cell conditioned medium and membrane proteins. (**B**) Heatmap revealing hierarchical clustering of secreted proteins recognized by B_{Vax}-derived Igs from CT2A conditioned medium. (**C**) Heatmap revealing hierarchical clustering of membrane proteins recognized by B_{Vax}-derived Igs from CT2A membrane proteins. Each triplicate corresponds to an independent IP-MS experiment. In Triplicate 1, B_{Vax}-derived Igs were pooled from 10 mice, and B_{Naive}-derived Igs were pooled from 11 mice. In Triplicate 2, B_{Vax}-derived Igs were pooled from 11 mice, and B_{Naive}-derived Igs were pooled from 10 mice. Triplicate 3 involved B_{Vax}-derived Igs pooled from 10 mice and B_{Naive}-derived Igs pooled from 12 mice.

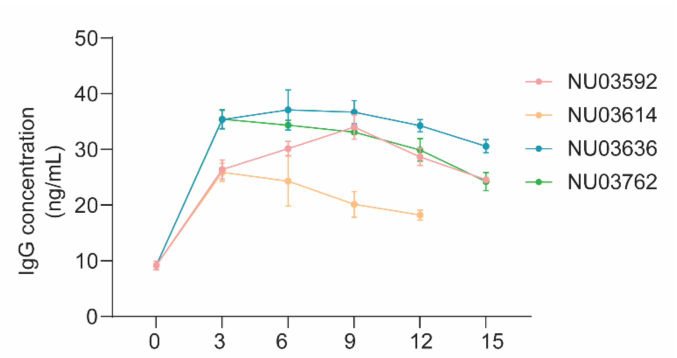

Supplementary Figure 4. Quantification of patient B_{Vax}-derived Igs. Supernatants from GBM patient B_{Vax} cells cultured in StemCell plasmablast differentiation medium were collected every 3 days. n=4. The presence of secreted IgG was confirmed by ELISA.

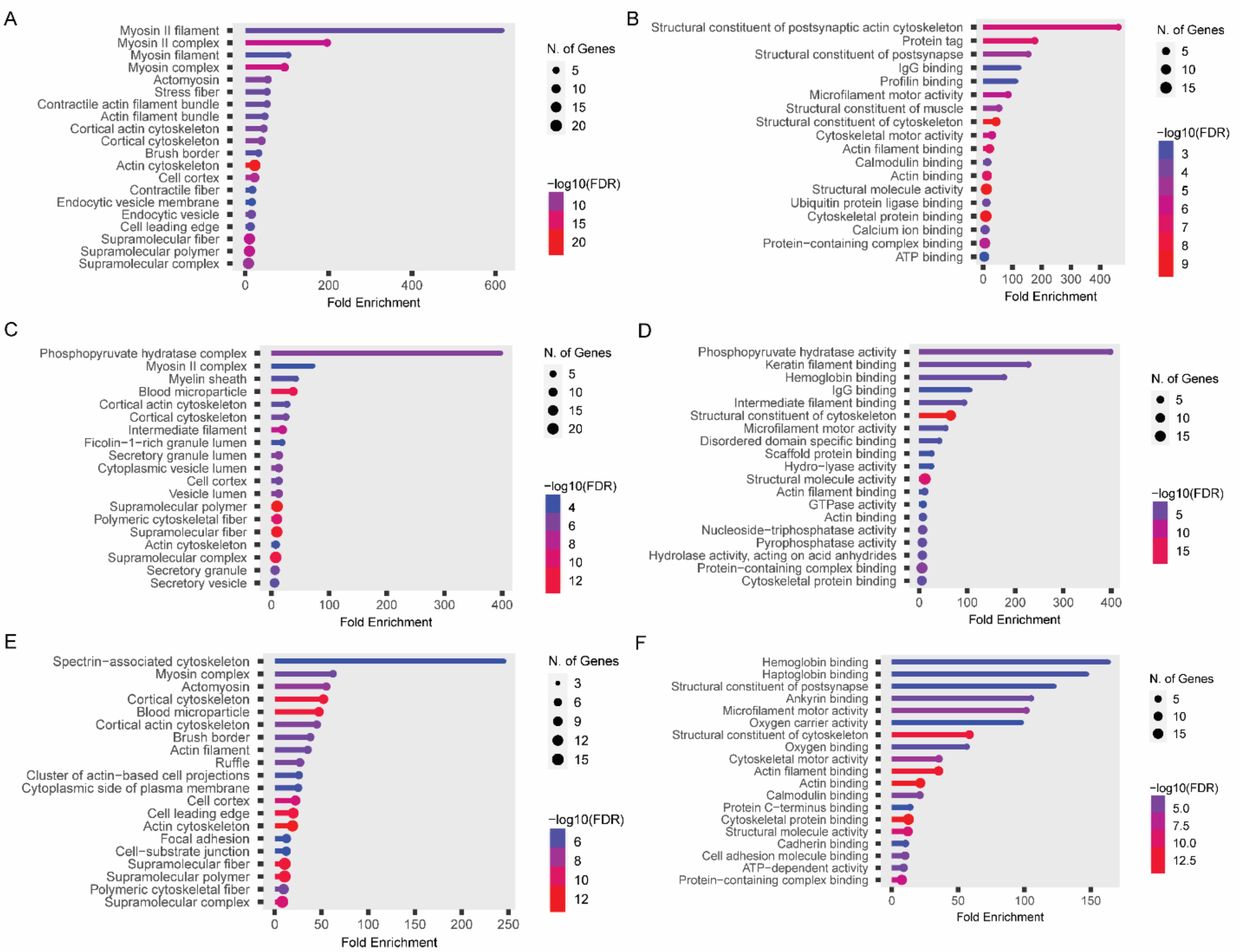

Supplementary Figure 5. Gene Ontology (GO) analysis of proteins recognized by human B_{Vax} produced Igs. False discovery rate (FDR) of GO analysis was acquired from ShinyGO 0.77. $p < 0.05$. (**A, C** and **E**) Top 20 GO cellular component terms from 3 replicates (NU02545, NU02569 and NU02594 respectively). (**B, D** and **F**) Top 20 GO molecular function terms from 3 replicates (NU02545, NU02569 and NU02594 respectively).

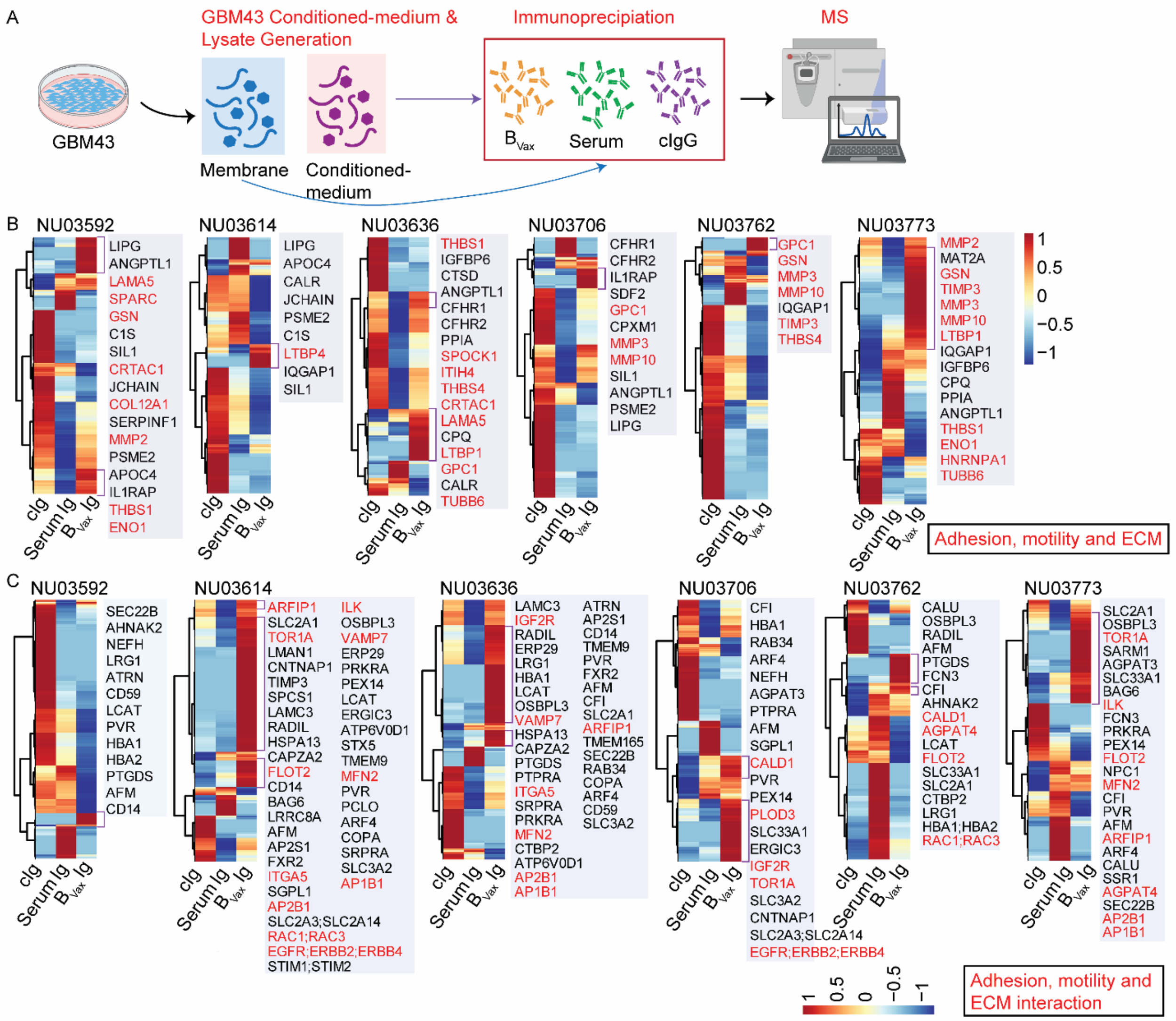

Supplementary Figure 6. Characterization of GBM patient B$_{Vax}$-derived Ig reactivity. (**A**) Schema depicting the protocol for the human IP-MS experiments used to identify tumor-specific antigens recognized by B$_{Vax}$-derived antibodies from GBM43 tumor cell conditioned medium and membrane proteins. (**B**) Heatmap revealing hierarchical clustering of secreted proteins recognized by B$_{Vax}$-derived Igs from GBM43 conditioned medium. n=6. (**C**) Heatmap revealing hierarchical clustering of membrane proteins recognized by B$_{Vax}$-derived Igs from GBM43 membrane proteins. n=6. Targets related to adhesion, motility, or the extracellular matrix (ECM)/ ECM interaction are shown in red.

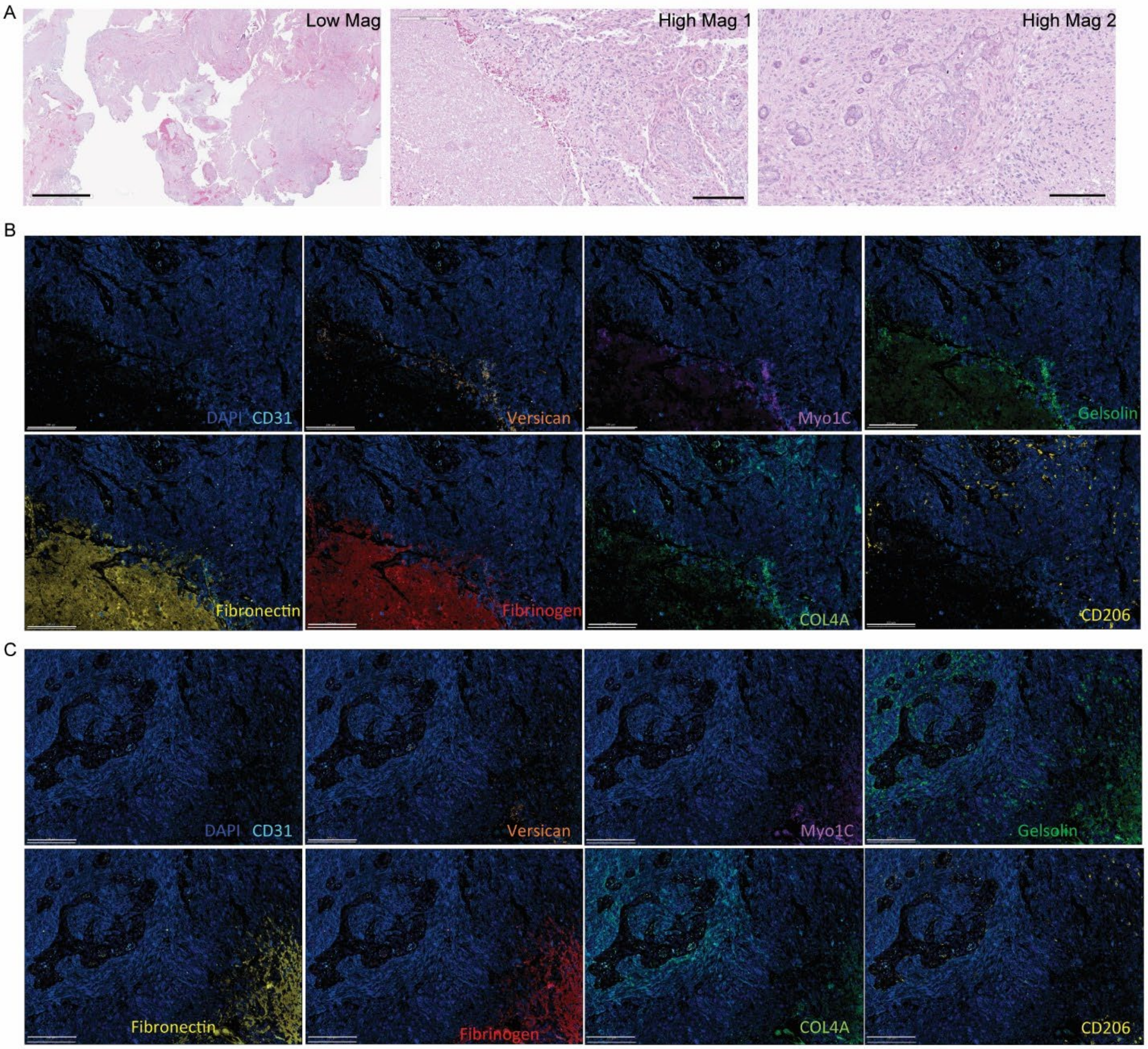

Supplementary Figure 7. B_{Vax}-derived Igs-recognized antigens are part of ECM from a paired GBM patient (NU02545). (**A**) Representative H&E staining images from patient NU02545 brain sections. Low Mag corresponds to Figure 5A (scale bar = 2mm); High Mag 1 corresponds to Supplementary Figure 7B (scale bar = 200μm); High Mag 2 corresponds to Supplementary Figure 7C (scale bar = 200μm). (**B-C**) Representative Spatial Multiplex immunofluorescence images generated using the COMET^{TM} system (Lunaphore Technologies) showing B_{Vax}-derived Igs-recognized antigens are part of GBM extracellular matrix (ECM). Scale bars = 200μm.

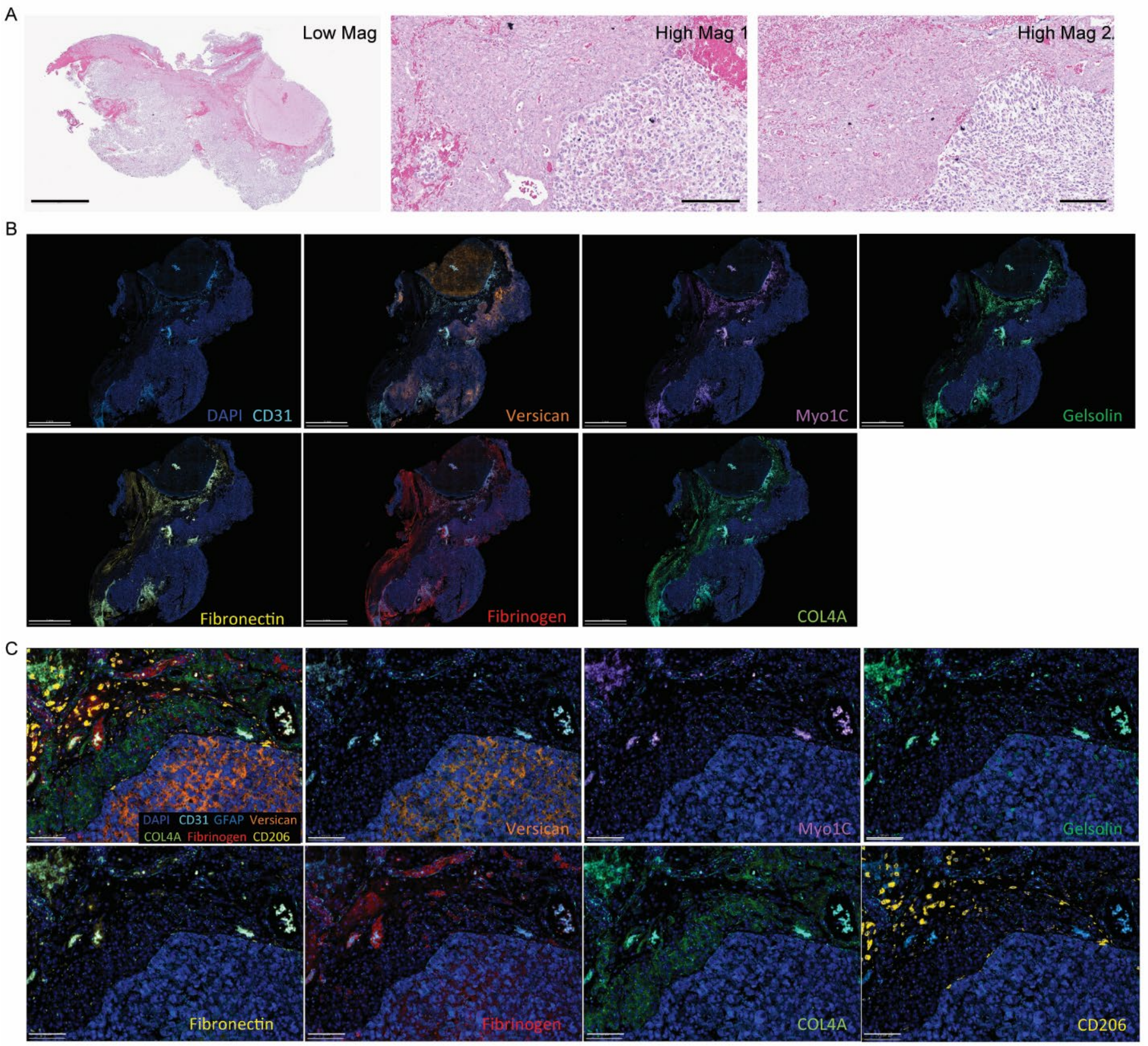

Supplementary Figure 8. B_{Vax}-derived Igs-recognized antigens are part of ECM from a paired GBM patient (NU02594). (**A**) Representative H&E staining images from patient NU02594 brain sections. Low Mag 1 corresponds to Supplementary Figure 8B that is flipped 90° (scale bar = 2mm); High Mag 1 corresponds to Supplementary Figure 8C (scale bar = 200μm); High Mag 2 corresponds to Figure 5B (scale bar = 200μm). (**B-C**) Representative Spatial Multiplex immunofluorescence images generated using the COMET^{TM} system (Lunaphore Technologies) showing B_{Vax}-derived Igs-recognized antigens are part of GBM extracellular matrix (ECM). (B) Scale bars = 2mm; (C) scale bars = 100μm.

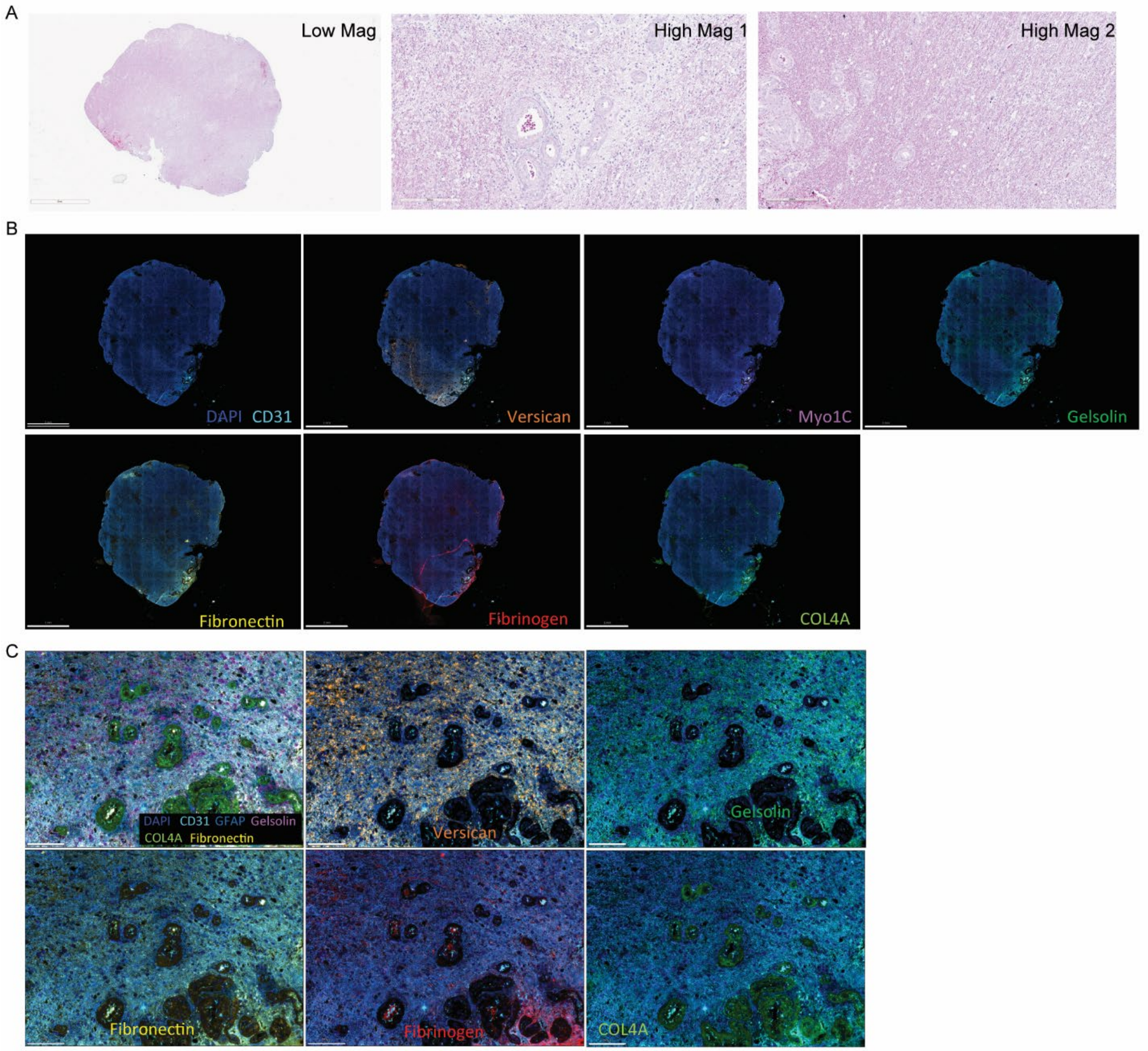

Supplementary Figure 9. B$_{Vax}$-derived Igs-recognized antigens are part of ECM from a paired GBM patient (NU02569). (**A**) Representative H&E staining images from patient NU02569 brain sections. Low Mag corresponds to Supplementary Figure 9B that is flipped 90° (scale bar = 2mm); High Mag 1 corresponds to Figure 5C (scale bar = 200μm); High Mag 2 corresponds to Supplementary Figure 9C (scale bar = 200μm). (**B-C**) Representative Spatial Multiplex immunofluorescence images generated using the COMET™ system (Lunaphore Technologies) showing B$_{Vax}$-derived Igs-recognized antigens are part of GBM extracellular matrix (ECM). (B) Scale bars = 2mm; (C) Scale bars = 100μm.

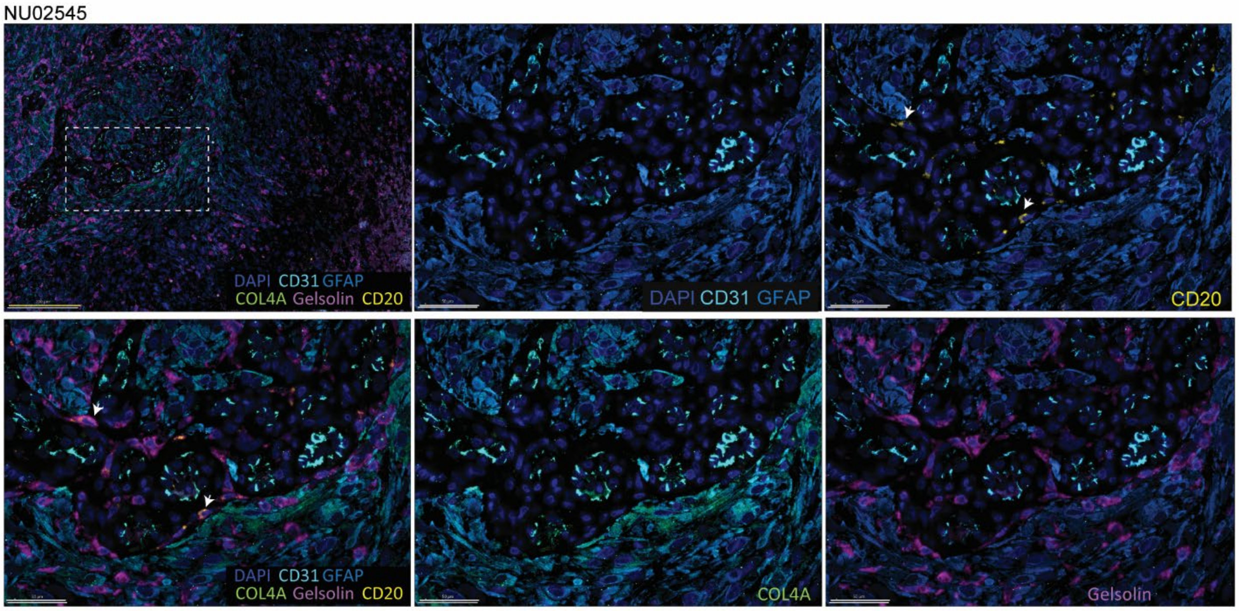

Supplementary Figure 10. B cells are present in the tumor region near B_{Vax}-derived Igs-recognized antigens. Representative Spatial Multiplex immunofluorescence images generated using the COMET™ system (Lunaphore Technologies) showing the presence of B cells (white arrowhead) in the tumor region. The left panel shows a low-magnification overview with a white box indicating the region of interest, which is shown at higher magnification in the subsequent panels. White dotted box shows where the high mag images were taken. Yellow scale bar = 200μm, white scale bar = 50μm.

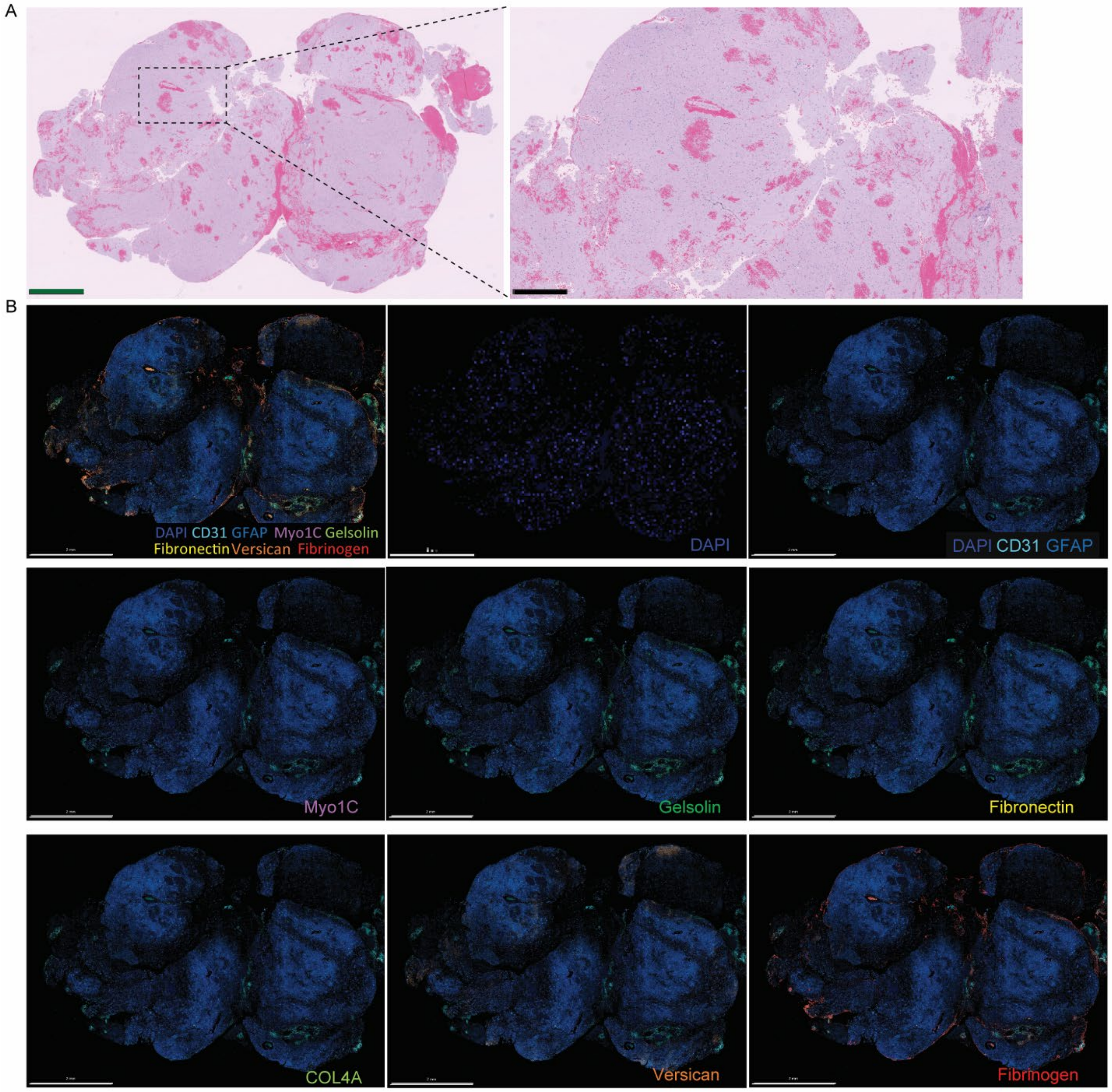

Supplementary Figure 11. Peritumoral brain shows a low expression of B_{Vax}-derived Igs-recognized antigens. (**A**) Representative H&E staining image of the peritumoral brain from a GBM patient. Green scale bar = 1mm, black scale bar = 250μm. (**B**) Representative Spatial Multiplex immunofluorescence images generated using the COMET™ system (Lunaphore Technologies) showing the intensity of B_{Vax}-derived Igs-recognized antigens in the peritumoral brain from a GBM patient (tumor was not included). Scale bar = 2mm.

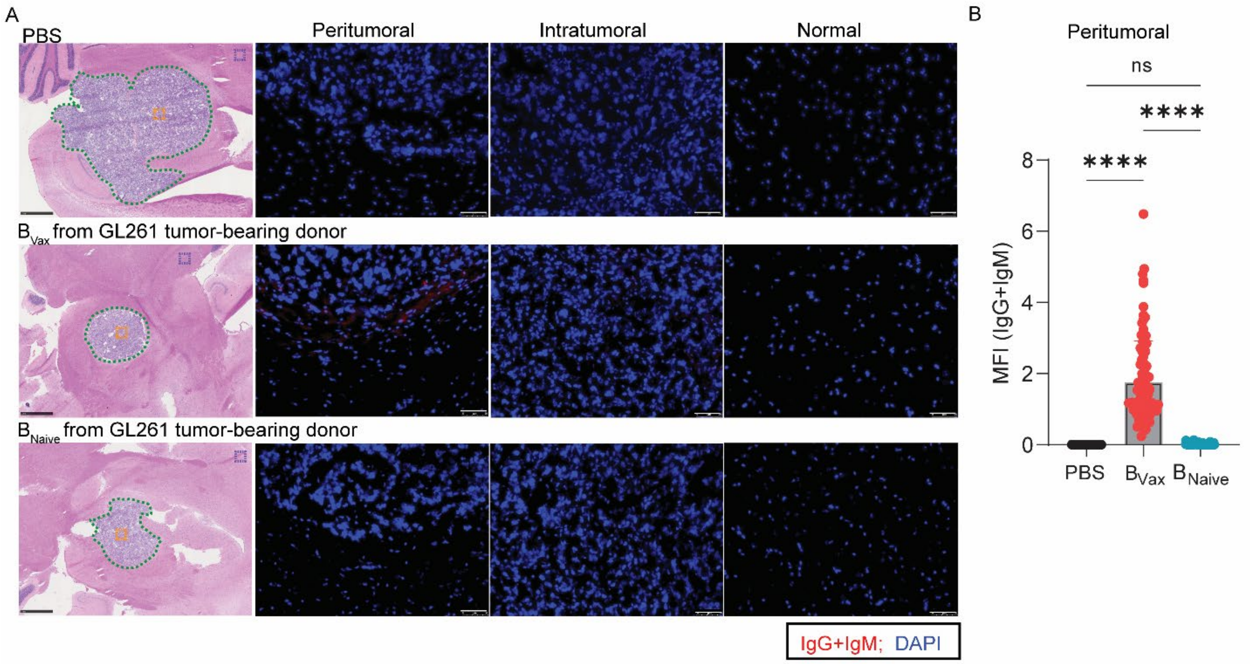

Supplementary Figure 12. B$_{Vax}$-Igs consistently localize to the peritumoral region in the GL261 GBM murine model. (**A**) Representative images of H&E and immunofluorescence (IF) staining for anti-mouse IgG and IgM to assess the presence and localization of B$_{Vax}$-derived antibodies. B$_{Vax}$ or B$_{Naive}$ cells from GL261 tumor-bearing C57BL/6 mice were adoptively transferred into GL261 tumor-bearing muMT (B cell knockout) mice. Following treatment, brain tissues were harvested from recipient mice and stained for anti-mouse IgG and IgM (red). H&E images show the organization of the tumors and the locations where the IF images were taken: peritumoral region (dotted green line), intratumoral region (orange box), and relatively normal brain (purple box). (**B**) Quantification of the relative intensity of B$_{Vax}$-Igs in the peritumoral region. 10-15 images were taken around the peritumoral region of each mouse (dotted green line). Mean Fluorescence Intensity (MFI) of anti-mouse IgG and IgM (red) in each image were quantified using ImageJ as described previously (69, 70). PBS group: n=3; B$_{Vax}$ group: n= 4; B$_{Naive}$ group: n= 3. Data are presented as the mean ± s.d. and were analyzed by one-way ANOVA. Statistical significance is indicated as follows: ns represents no significance, and ****$P < 0.0001$.
